# SAS-1 Is a C2 Domain Protein Critical for Centriole Integrity in *C. elegans*

**DOI:** 10.1371/journal.pgen.1004777

**Published:** 2014-11-20

**Authors:** Lukas von Tobel, Tamara Mikeladze-Dvali, Marie Delattre, Fernando R. Balestra, Simon Blanchoud, Susanne Finger, Graham Knott, Thomas Müller-Reichert, Pierre Gönczy

**Affiliations:** 1 Swiss Institute for Experimental Cancer Research (ISREC), School of Life Sciences, Swiss Federal Institute of Technology (EPFL) Lausanne, Switzerland; 2 Friedrich Miescher Institute for Biomedical Research, Basel, Switzerland; 3 University of Basel, Basel, Switzerland; 4 BioEM Facility, School of Life Sciences, Swiss Federal Institute of Technology (EPFL) Lausanne, Switzerland; 5 Structural Cell Biology Group, Experimental Center, Medical Faculty Carl Gustav Carus, Technische Universität Dresden, Dresden, Germany; Washington University School of Medicine, United States of America

## Abstract

Centrioles are microtubule-based organelles important for the formation of cilia, flagella and centrosomes. Despite progress in understanding the underlying assembly mechanisms, how centriole integrity is ensured is incompletely understood, including in sperm cells, where such integrity is particularly critical. We identified *C. elegans sas-1* in a genetic screen as a locus required for bipolar spindle assembly in the early embryo. Our analysis reveals that sperm-derived *sas-1* mutant centrioles lose their integrity shortly after fertilization, and that a related defect occurs when maternal *sas-1* function is lacking. We establish that *sas-1* encodes a C2 domain containing protein that localizes to centrioles in *C. elegans*, and which can bind and stabilize microtubules when expressed in human cells. Moreover, we uncover that SAS-1 is related to C2CD3, a protein required for complete centriole formation in human cells and affected in a type of oral-facial-digital (OFD) syndrome.

## Author Summary

Centrioles are microtubule-based organelles critical for forming cilia, flagella and centrosomes. Centrioles are very stable, but how such stability is ensured is poorly understood. We identified *sas-1* as a component that contributes to centriole stability in *C. elegans*. Centrioles that lack *sas-1* function loose their integrity, and our analysis reveals that *sas-1* is particularly important for sperm-derived centrioles. Moreover, we show that SAS-1 binds and stabilizes microtubules in human cells, together leading us to propose that SAS-1 acts by stabilizing centriolar microtubules. We identify C2CD3 as a human homolog of SAS-1. C2CD3 is needed for the presence of the distal part of centrioles in human cells, and we thus propose that this protein family is broadly needed to maintain centriole structure.

## Introduction

Centrioles are small microtubule-based organelles that are critical for the formation of cilia and flagella across eukaryotic evolution, as well as for that of centrosomes in animal cells. Centriolar microtubules exhibit unusual stability, which is thought to contribute to the integrity of the entire organelle. That centrioles retain such integrity is probably key not only to withstand mechanical stresses generated by cilia, flagella and centrosomes, but also to ensure proper assembly of new centrioles in proliferating cells.

Several components important for centriole assembly have been uncovered in the last decade (reviewed in [1], [2]). In *C. elegans*, genetic and functional genomic screens have led to the identification of five core components that act sequentially during centriole biogenesis [3]–[10]. The first protein to be recruited to centrioles is SPD-2, which is required for the subsequent presence of the kinase ZYG-1 [11], [12]. The next components to be loaded onto centrioles are the interacting proteins SAS-6 and SAS-5. At this stage, a central tube can be observed by electron microscopy as the first emerging structure during centriole biogenesis [12]. Next, SAS-4 is incorporated, after which centriolar microtubules are added onto the forming organelle. Centrioles in *C. elegans* are only ∼100 nm in both length and width, and are comprised of microtubule singlets [5], [13]. Due to their minute size, pairs of centrioles in *C. elegans* cannot be resolved by immunofluorescence analysis, except after their disengagement from one another at the end of mitosis [8], [10]. Some features of *C. elegans* centrioles differ from those in most other systems, where centrioles are typically ∼450 nm in length and ∼250 nm in diameter and harbor microtubule triplets [14], [15]. Nevertheless, homologs of the core components initially identified in worms turned out to be critical for centriole formation from algae to humans [16], [17]. This indicates that *C. elegans* can serve as a model to discover fundamental and conserved features of centriole biology.

In contrast to most cytoplasmic microtubules that exhibit dynamic instability [18], [19], centriolar microtubules are very stable, resisting cold- and nocodazole-induced microtubule depolymerization [20]. Accordingly, microtubules of centrioles from human cells purified at 4°C are comparable by electron microscopy to those of centrioles in cells [15], [21], [22]. Moreover, pulse-chase experiments with labeled α- and β-tubulin subunits demonstrated that centriolar microtubules exhibit little turnover over one cell cycle in vertebrate tissue culture cells [23]. The α-tubulin subunit of centriolar microtubules undergoes substantial post-translational modifications, including acetylation and de-tyrosinatation [20], [24], [25], as well as polyglutamylation, which also occurs on the β-tubulin subunit of centriolar microtubules [26]–[28]. Some of these modifications, in particular polyglutamylation, appear to not only mark stable microtubules, but also to contribute to their stability [29], [30]. Accordingly, injection of antibodies against polyglutamylated tubulin leads to loss of centriole integrity in human cells [31].

Several proteins have been identified as being important for the stabilization of centriolar microtubules in human cells, including hPoc1, CAP350 and centrobin [32]–[35]. Poc1 is an evolutionarily conserved protein that associates with microtubules *in vitro* and localizes notably to centriolar microtubules *in vivo* [33], [36], [37]. Depletion of Poc1 from human cells or its inactivation in *Drosophila* results in shorter centrioles, whereas overexpression leads to overly long centriole-like structures [38], [39]. Moreover, depletion of Poc1 in *Tetrahymena* leads to the formation of basal bodies that have compromised integrity, being more sensitive to nocodazole [33]. Likewise, centrioles in human cells exhibit nocodazole sensitivity when depleted of CAP350, a microtubule-associated protein that localizes to centrioles [32]. Centrobin, a component present solely in newly formed centrioles, interacts with tubulin *in vitro* and *in vivo*, and this interaction is needed for centriole stabilization [34], [40]. These studies notwithstanding, how the unusual long-term stability of centrioles is achieved remains incompletely understood, especially in the context of a developing organism.

A particularly acute need for centriole stability is encountered during spermatogenesis in many species, since the centrioles that are formed during the meiotic divisions of male germ cells must be retained during the entire course of spermiogenesis to then be contributed to the zygote (reviewed in [41]). By contrast, centrioles are eliminated or inactivated during oogenesis. As a result, the newly fertilized embryo is endowed strictly with paternally contributed centrioles. Next to these original centrioles, new centrioles are then assembled during the first cell cycle with maternally provided components. For these events to occur faithfully, paternally contributed centrioles must retain their integrity throughout spermatogenesis and after fertilization. Despite such retention of centriole integrity being critical for embryonic development, the underlying mechanisms remain to be discovered.

## Results

### *sas-1* is required for bipolar spindle assembly

**Figure 1:**
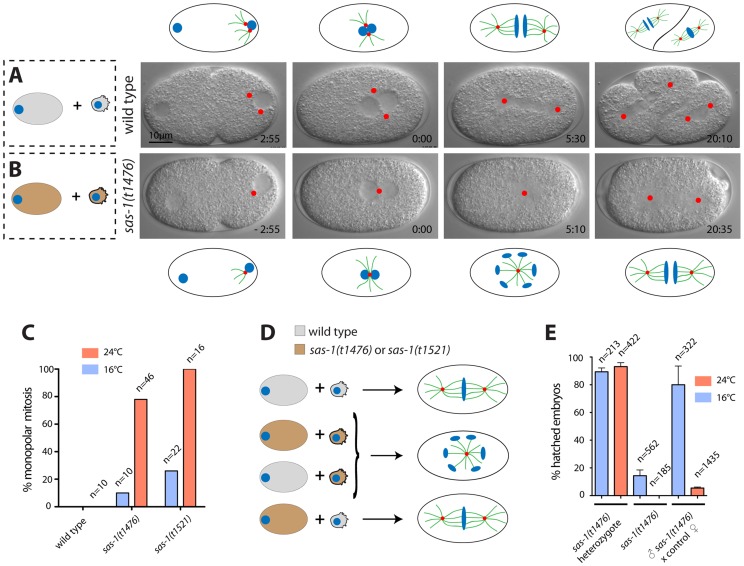
*sas-1* is a paternal-effect mutation required for bipolar spindle assembly in the embryo. (A–B) Images from DIC time-lapse recording of wild type (A) or *sas-1(t1476)* (B) embryo. In this and other panels, time is indicated in min and sec, with t = 0:00 corresponding to pronuclear meeting. Red dots indicate MTOCs. See Movies S1 and S2. Schematics are shown to help interpret the corresponding DIC images. (C) Percentage of embryos of the indicated genotypes exhibiting monopolar spindle assembly in the first cell cycle. Note that the wild type was only imaged at 24°C. (D) Schematic representing the typical phenotype of one-cell stage embryos resulting from the joining of the indicated gametes. (E) Progeny test of indicated conditions. Although heterozygote *sas-1(t1476)* are not 100% viable, this likely reflects experimental variability, as the effect is weaker at the restrictive temperature. See also Table S1 and Fig. S1–S3.

The *sas-1* (spindle assembly abnormal 1) locus was identified in a screen for parental-effect embryonic lethal mutations [42]. Using time-lapse DIC microscopy, we found that embryos derived from *sas-1(t1476)* or *sas-1(t1521)* homozygote mutant animals raised at 24°C almost invariably assemble a monopolar spindle in the first cell cycle (Fig. 1A–E, Movies S1–S4, Table S1). In the second cell cycle, while most of the embryos then assemble a bipolar spindle, some exhibit monopolar or tripolar spindle assembly (Table S1). Both mutant alleles are temperature-sensitive, as evidenced by spindle assembly in the first cell cycle being bipolar in the majority of cases at 16°C (Fig. 1C). To test whether *sas-1(t1476)* behaves as a null allele at 24°C, we crossed it to a strain carrying the deficiency *eDf2*, in which the large region of chromosome III to where *sas-1* had been located is missing [42]. We again observed monopolar spindle assembly in the resulting embryos in the first cell cycle (Movie S5, Table S1), as well as sterility in some of the animals, which is never observed in *sas-1(t1476)* homozygous animals. Together, these findings indicate that *sas-1(t1476)* is a severe reduction-of-function allele, but not a null. For historical reasons, we focused further analysis on *sas-1*(*t1476*), but found similar results to the ones reported below with *sas-1*(*t1521*) (Table S1). Unless stated otherwise, we will use the term “*sas-1* mutant” hereafter to refer to *sas-1(t1476)* homozygous animals.

We tested whether centriolar and pericentriolar material (PCM) components are present in the monopolar spindle assembled in *sas-1* mutant embryos. To this end, we conducted immunofluorescence analysis with antibodies against the centriolar proteins SAS-4, SAS-5, SAS-6 and IFA, as well as the PCM proteins SPD-5 and SPD-2, the latter also marking centrioles. We also labeled microtubules in these experiments using antibodies against α-tubulin to determine the number of microtubule organizing centers (MTOCs). As anticipated from the results with time-lapse DIC microscopy, this analysis revealed that a large majority of *sas-1* mutant embryos at pronuclear migration/pronuclear meeting (35/40) and during mitosis (42/55) harbor a single MTOC that contains centriolar and PCM components (Fig. S1 and Table S2). In addition, we found embryos in which MTOCs were devoid of centriolar proteins (Table S2). Furthermore, in agreement with the occasional tripolar spindles observed by time-lapse microscopy in the second cycle, we also observed some tripolar configurations by immunofluorescence analysis, with the three spindle poles usually exhibiting different sizes (Table S2). To investigate the origin of tripolar spindles, we generated a *sas-1* mutant strain expressing the centriolar marker GFP-SAS-6 and the microtubule marker mCherry-β-tubulin. Of the nine embryos analyzed in which a tripolar spindle assembled in the second cell cycle, we found that in four cases, all three spindle poles harbored GFP-SAS-6 throughout the recording (see Fig. S2). In the remaining five embryos, at least one of the three spindle poles marked by mCherry-β-tubulin did not harbor GFP-SAS-6 by the time of mitosis. Accordingly, immunofluorescence analysis confirmed the presence of occasional MTOCs devoid of centriolar markers (Table S2). We conclude that tripolar spindle assembly is often directed by spindle poles that lack the centriolar marker GFP-SAS-6, but retain MTOC activity.

### Paternal *sas-1* is required for bipolar spindle assembly in the first embryonic cycle

To test whether monopolar spindle assembly in the first cell cycle reflects a paternal and/or a maternal requirement, *sas-1* mutant males were mated with wild type hermaphrodites. Time-lapse DIC microscopy revealed monopolar spindle assembly in the first cell cycle in ∼80% of the resulting embryos (Table S1). Moreover, we found that ∼95% of the progeny of *sas-1* mutant males mated with control hermaphrodites do not hatch (Fig. 1E). We conclude that *sas-1* has a strong paternal requirement.

One possible explanation for monopolar spindle assembly in the first cell cycle followed by bipolar spindle assembly in the majority of embryos in the second cell cycle is that *sas-1* mutant sperm harbors one centriole instead of the usual two, as in embryos derived from males mutant for *zyg-1* or *sas-5* [5], [6]. To test this possibility, we performed serial section electron microscopy of high pressure frozen sperm cells. Although we cannot exclude that more subtle defects have gone unnoticed, this analysis revealed that *sas-1* mutant sperm contain two centrioles with detectable microtubule blades, analogous to the situation in the wild type (Fig S3A–B). Moreover, immunofluorescence analysis demonstrated that *sas-1* mutant sperm harbor SAS-4, SAS-5 and SAS-6 (Fig. S3C–I). Together, these results establish that although there is a paternal requirement for *sas-1* function, mutant sperm cells contain two centrioles that do not seem different from the wild type.

### *sas-1* mutant paternal centrioles are unstable

**Figure 2:**
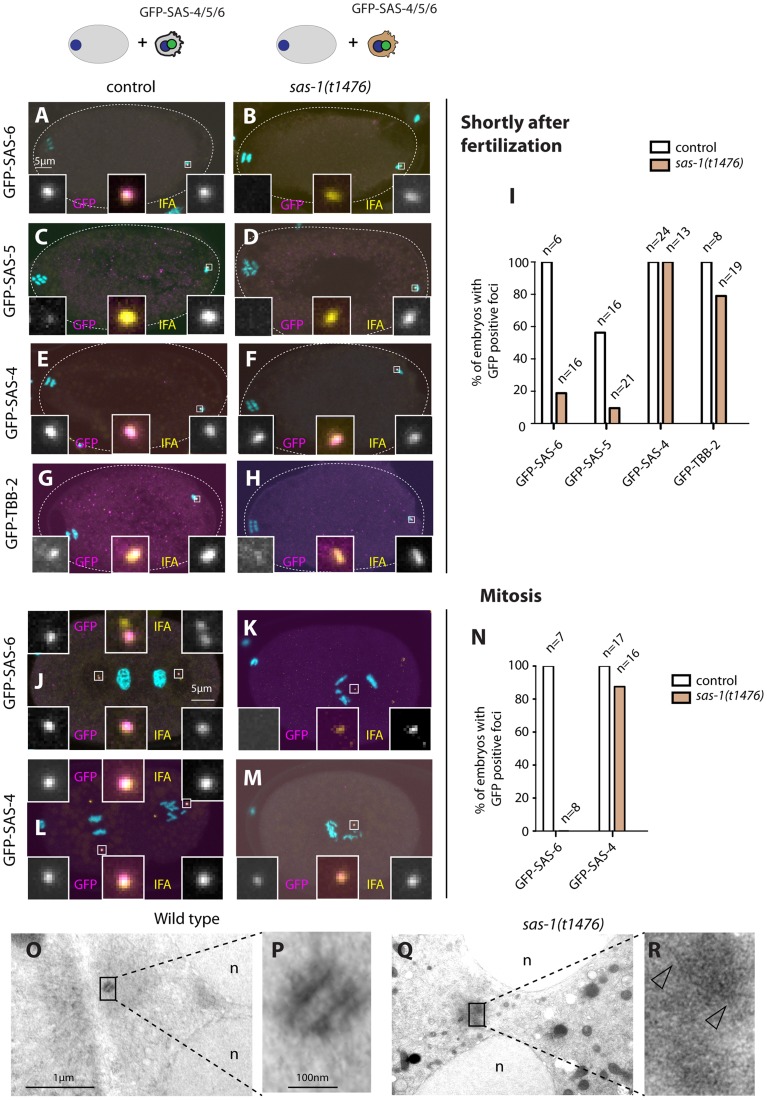
*sas-1* is required paternally for centriole integrity in the embryo. (A–H, J–M) *sas-1(t1476)* heterozygote (control, A, C, E, G, J, L) or *sas-1(t1476)* homozygote (B, D, F, H, K, M) males expressing the indicated GFP fusion proteins were mated to control animals and the resulting embryos analyzed just after fertilization (A–H) or during mitosis (J–M), staining for GFP (magenta) and IFA (yellow). DNA is shown in cyan. In this and other figures, insets are ∼6 fold magnified views of boxed regions unless stated otherwise. (I, N) Quantification of experiments shown in A–H (I) and J–M (N). (O–R) Electron micrograph of a wild type centriole (O–P) and of a serially-sectioned *sas-1(t1476)* mutant embryo at pronuclear meeting (Q–R). Note electron dense material in the *sas-1* mutant embryo, with no recognizable centriolar cylinder. 3 *sas-1(t1476)* embryos were analyzed and no centrioles were found. Note also microtubules emanating from this area (arrowheads). See also Fig. S4–S5.

What happens to the seemingly normal centrioles contributed by *sas-1* mutant sperm once in the embryo? To address this question, we followed the fate of paternal centrioles labeled by GFP-SAS-6, GFP-SAS-4 or GFP-β-tubulin (Fig. 2A–N). To this end, males expressing these fusion proteins were mated with control hermaphrodites, and the resulting embryos analyzed shortly after fertilization, as well as at the end of the first cell cycle.

Shortly after fertilization, we found that whereas 100% of control embryos retain paternal GFP-SAS-6, the signal is present in only ∼20% *sas-1* mutant embryos (Fig. 2A–B, I). The same holds for endogenous SAS-6 (Fig. S4A). We found a similar trend with GFP-SAS-5, although the outcome is less telling in this case because SAS-5 exchanges readily with the cytoplasmic pool shortly after fertilization even in the wild type [6] (Fig. 2C–D, I). There is also a slight diminution in the case of GFP-β-tubulin, with ∼80% of centrioles contributed by *sas-1* mutant sperm exhibiting a GFP focus, as opposed to 100% in the control condition (Fig. 2G–H, I). In the case of GFP-SAS-4, all embryos in both control and *sas-1* mutant condition retain focused signals shortly after fertilization (Fig. 2E–F, I). This experiment also allowed us to conclude that there is no defect in centriole disengagement in *sas-1* mutants, since two GFP-SAS-4 foci can be distinguished shortly after fertilization in ∼30% of embryos fertilized by either control or *sas-1* mutant sperm (see also Fig. S4B–D for endogenous SAS-4).

Next, we examined the fate of paternal centrioles marked by GFP-SAS-6 or GFP-SAS-4 during mitosis, at the end of the first cell cycle. Whereas both proteins are invariably present as two foci in control conditions, we found that GFP-SAS-6 is never present when originating from *sas-1* mutant sperm (Fig. 2J–K). Moreover, we found that a single focus of paternal GFP-SAS-4 is detected during mitosis in almost all *sas-*1 mutant embryos analyzed (Fig. 2L–N). We conclude that one of the two paternally contributed centrioles disappears by the end of the first cell cycle, whereas the second looses GFP-SAS-6 but still harbors GFP-SAS-4. This conclusion is in line with the live imaging analysis of tripolar configurations in the second cell cycle that revealed loss of GFP-SAS-6 from some spindle poles (see Fig. S2).

The above results suggest that centrioles contributed by *sas-1* mutant sperm somehow loose stability after fertilization. To address whether this is accompanied by a detectable ultra-structural defect, we performed electron microscopy following high pressure freezing of *sas-1* mutant embryos in the first cell cycle. Intriguingly, we found that the characteristic microtubule-based structure of centrioles observed in the wild type (Fig. 2O–P) is no longer recognizable in *sas-1* mutant embryos (Fig. 2Q–R). Instead, we observed electron dense material in the area where centrioles should reside (Fig. 2R); microtubules can be seen emanating from this area (Fig. 2R, arrowheads), which presumably contains the focus of SAS-4 and α-tubulin detected by immunofluorescence. Overall, we conclude that centrioles loose their integrity and lack organized centriolar microtubules in *sas-1* mutant embryos.

### Paternally contributed centriolar structures can recruit new centriolar material in *sas-1* mutant embryos

Because *sas-1* mutant embryos typically form a bipolar spindle in the second cell cycle, we hypothesized that the centriolar structure contributed by *sas-1* mutant sperm that retains GFP-SAS-4 signal up to mitosis is sufficient to foster the formation of a centriole-like structure in its vicinity, even if it is not a canonical centriole as evidenced by electron microscopy analysis (see Fig. 2Q–R). To test this hypothesis, we crossed *sas-1* mutant males to control hermaphrodites expressing GFP-SAS-4 or GFP-SAS-6 (Fig. S5A–F). We found that GFP-SAS-4 and GFP-SAS-6 are recruited in the vicinity of the single paternally contributed centriolar structure that remains as embryos progress through the first cell cycle (Fig. S5A–F). The same holds for GFP-SAS-6 when both males and hermaphrodites are mutant for *sas-1* (Fig. S5G–J). We conclude that both SAS-6 and SAS-4 are recruited to the paternally contributed centriolar structure contributed by *sas-1* mutant sperm. This likely explains why *sas-1* mutant embryos usually undergo bipolar spindle assembly in the second cell cycle.

### Maternal *sas-1* is required for proper spindle formation during later cell cycles

**Figure 3:**
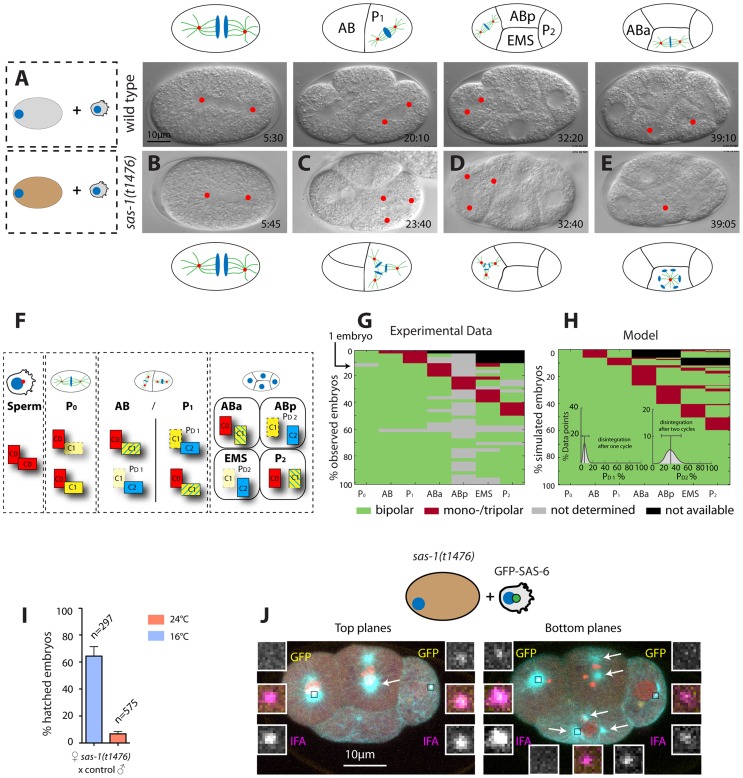
*sas-1* is required maternally for centriole integrity. (A–E) Images from DIC time-lapse recordings of a wild type embryo (A) and of four embryos resulting from the fertilization of a *sas-1(t1476)* oocyte by control sperm (B–E). See Movies S1 and S6. Note that in some instances tripolar spindles were observed, presumably because centrioles disintegrate during mitosis (see main text). (F) Schematic showing centriole duplication in the first two cell cycles. C0  =  centrioles contributed by sperm (red), C1  =  centrioles formed next to C0 centrioles (yellow), C2  =  centrioles formed next to C1 centrioles (blue). Blue lines in C1 centrioles indicate formation during the second cell cycle. See also (G–H). (G–H) Visualization of the actual division patterns (G, n = 40 movies) and model 2 (H), with 50’000 simulated embryos. Green  =  Bipolar division, red  =  mono- or tripolar division, black  =  not relevant (descendant of failed division), grey  =  not determined. Insets in (H): a mathematical model of centriole disintegration. This model 2 assumes a probability for the disintegration one cell cycle after C1 formation ( = P_D1_) and a different probability for disintegration two cell cycles after C1 formation ( = P_D2_). The most likely probabilities given the experimental data (see F) are P_D1_ = 0.0625 (0.0253–0.1385, 95% CI) and P_D2_ = 0.3028 (0.1465–0.4768, 95% CI). The data is from both *sas-1(t1476)* (N = 16, 6 at 24°C, 10 at 20°C) and *sas-1(t1521)* (N = 24, 14 at 24°C, 10 at 20°C) mated to either *fog-2* or *plg-1* males. (I) Progeny test revealing *sas-1* maternal requirement. (J) A *sas-1* mutant oocyte fertilized by wild type sperm carrying GFP-SAS-6- labeled centrioles, stained for tubulin (cyan), GFP (yellow) and IFA (magenta). DNA is shown in red. Shown are the top 10/20 planes and bottom 10/20 Z-planes; arrows point to the poles of the tripolar figures (note that one MTOC in ABp is present in both bottom and top planes and only indicated once). Note that whereas the tripolar figures are in ABp and EMS, the paternal GFP-SAS-6 positive centrioles are in ABa and P_2_; N = 8 embryos at the four-cell stage that exhibit a phenotype in at the least one blastomere. Given that there are 32 blastomeres in total and that 10 of them exhibited abnormal spindle assembly (6 embryos with one abnormal blastomere, 2 embryos with two abnormal blastomeres –ABp and EMS in one case, ABa and EMS in the other) and assuming that paternally contributed centrioles have a 50% chance of ending up in any blastomere, it follows that the likelihood that the absence of paternal centrioles in those blastomeres that exhibit abnormal spindle assembly is purely due to chance is 0.5^10^ = 9.7×10^−4^.

The fact that *sas-1* mutant males mated to control hermaphrodites give rise to 5% viable progeny whereas self fertilized *sas-1* mutants yield none indicates that there is also a maternal requirement for *sas-1*. In order to uncover the nature of this requirement, control males were mated to *sas-1* mutant hermaphrodites and the resulting progeny analyzed by time-lapse DIC microscopy. Interestingly, these embryos always assemble a bipolar spindle in the first cell cycle, but exhibit monopolar or tripolar spindle assembly in some blastomeres starting typically in the third cell cycle or thereafter (Fig. 3A–E, G Movie S6). We noted also that ∼7% of such embryos hatch (Fig. 3I), suggesting that in some cases the majority of divisions must have happened normally, perhaps reflecting the onset of zygotic transcription in these *sas-1*(*t1476*) heterozygous embryos.

Extrapolating from the phenotypic analysis of embryos endowed with centrioles from *sas-1* mutant sperm, these observations suggest that impairment of maternal *sas-1* function results in the loss of centriole integrity with some probability. To estimate this probability, we first developed a simple mathematical model where centrioles would disintegrate after their formation with a single probability inferred from the data. This model predicted a loss of centriolar integrity for 12.5% of the centrioles over the course of the experiment (Materials and Methods, model 1). However, this predicted percentage is higher than the rate of failure observed experimentally at the two cell stage (8.5%). Thus, we developed a second model in which the probability of loosing centriole integrity is allowed to differ depending on the age of the centriole (Materials and Methods, model 2). Using a maximum-likelihood optimization procedure to identify the most probable values given the experimental data, we found the probability of losing centriole integrity one cell cycle after its formation to be 6.3% and two cell cycles after its formation to be 30.3% (Fig. 3F–H, Materials and Methods, model 2). Importantly, this model fit our data significantly better than the first model (p = 0.02, likelihood ratio test).

As can be seen in Fig. 3F (right-most box), this model predicts that in embryos lacking solely maternal *sas-1* function, those blastomeres in four-cell stage embryos that inherit one of the two centrioles contributed by wild type sperm should invariably exhibit bipolar spindle assembly. By contrast, those blastomeres that do not have paternally provided wild type centrioles could exhibit abnormal spindle assembly. To test this prediction, we fertilized *sas-1* mutant oocytes with wild type sperm harboring GFP-SAS-6 positive centrioles and analyzed the resulting embryos at the four-cell stage. This revealed that paternally contributed centrioles are never present in those cells that exhibit abnormal spindle assembly (Fig. 3J, 0/8 embryos). Overall, we conclude that upon compromised maternal *sas-1* function, centriole formation is initiated but the resulting structure looses integrity over time.

### SAS-1 is a C2 domain containing protein that localizes to centrioles

**Figure 4:**
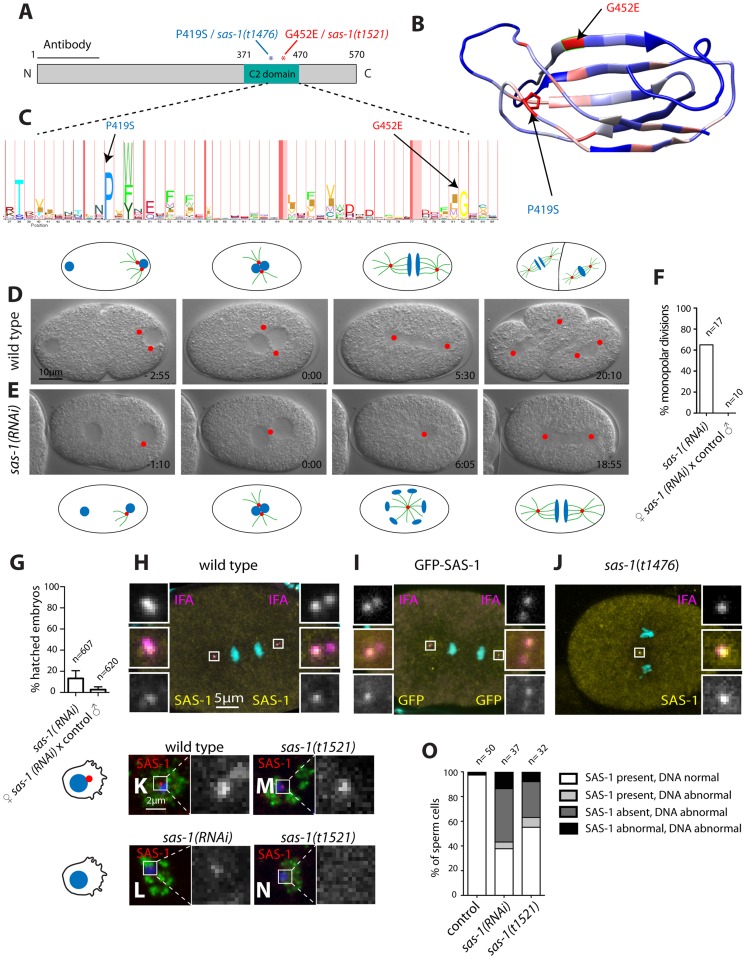
SAS-1 is a C2 domain containing protein that localizes to centrioles. (A) Schematic of SAS-1 protein architecture with an indication of the single amino acid substitutions in the two *sas-1* alleles, and the part against which the antibody was raised. (B) 3D model of the predicted C2 domain of SAS-1 (aa 371–470). Coloring of the residues is according to conservation (blue least, red most). The two amino acids mutated in the *sas-1* alleles are indicated. (C) Hidden Markov Model (HMM) representation of the C2 domain, obtained from Pfam (PF00168) [78]. (D–E) Images from DIC time-lapse recordings of wild type (D) and *sas-1(RNAi)* (E) embryo. (F) Quantification of *sas-1(RNAi)* phenotype by time-lapse DIC microscopy. (G) Progeny test revealing the paternal requirement for *sas-1(RNAi)*. The two experiments were not from the same batch of RNAi plates, likely explaining the lower viability in the progeny of the mated animals. (H–J) Immunostainings of a GFP-SAS-1 embryo for GFP (yellow) and IFA (magenta) (I), as well as of a wild type (H) or *sas-1*(*t1476*) (J) embryo stained for SAS-1 (yellow) and IFA (magenta). DNA is in cyan in all panels. (K–N) Wild type (K), *sas-1(RNAi)* (L) and *sas-1(t1521)* (M–N) sperm cells stained with the sperm marker SP-56 (green) and SAS-1 (red and shown alone in magnified panels). DNA is in blue. Note that a signal is present in (M), while it is absent in (N). Note that in (O), SAS-1 abnormal indicates that SAS-1 is either absent or, alternatively, present in too many foci, which we interpret to reflect meiotic defects and/or centrioles in the process of disintegrating. Note also that in *sas-1(RNAi)*, ∼6% of sperm cells do not harbor SAS-6, indicating that under those conditions, some sperm cells may be without centrioles altogether. Note that these experiments were performed with *sas-1(t1521)*. (O) Quantification of experiments shown in (K–N). See also Fig. S6.

Using SNP mapping and whole genome sequencing, we mapped the *sas-1* locus to the ORF Y111B2a.24 (Materials and Methods). This locus encodes a 570 amino acid (aa)-long protein predicted to contain a C2 domain (Fig. 4A). C2 domains have been implicated in membrane targeting, calcium binding and protein-protein interactions (reviewed in [43]). The two *sas-1* alleles harbor single amino acid substitutions at conserved residues within this C2 domain: *sas-1*(*t1476*) a P419S alteration and *sas-1*(*t1521*) a G452E change (Fig. 4A). Importantly, both residues are conserved in a variety of C2 domains from different phyla (Fig. 4B–C), indicating their importance for function.

To verify that the correct locus has been identified, we generated a strain expressing GFP-SAS-1 and found that this fusion protein can rescue *sas-1(t1476)* mutant embryos (Fig. S6A). The rescue is of ∼64% likely because the *gfp-sas-1* transgene is driven from the maternal *pie-1* promoter instead of the endogenous one (Fig. S6A). Mating *sas-1*(*t1476*) GFP-SAS-1 males to control hermaphrodites results in ∼15% viability, indicating partial paternal rescue, whereas mating *sas-1* homozygote males to *sas-1*(*t1476*) GFP-SAS-1 hermaphrodites results in ∼32% viability, indicating partial maternal rescue (Fig. S6A). Overall, we conclude that the ORF Y111B2a.24 indeed encodes SAS-1.

Having ascertained the identity of the *sas-1* locus, we performed RNAi to investigate whether a more severe phenotype could be revealed. When injecting animals with *sas-1* dsRNA to deplete the maternal pool using RNAi, we observed by immunofluorescence the presence of multipolar spindles, starting typically at the four-cell stage, just like for embryos maternally mutant for *sas-1* (Fig. S6B–C, compare to Fig. 3A–E, 3J). Furthermore, we sought to deplete both paternal and maternal pools by subjecting animals to RNAi from the first instar larval stage onwards (Materials and Methods). We found that some of the resulting *sas-1(RNAi)* animals are sterile, in line with the data acquired using the *eDf2* deficiency (see Table S1). Moreover, we found that ∼65% of *sas-1*(*RNAi*) embryos assemble a monopolar spindle during the first cell cycle (Fig. 4D–F, N = 17, Movie S7). From this subset, ∼73% exhibit a more severe phenotype than that usually observed in the first cell cycle of *sas-1* mutant embryos, with the two pronuclei migrating only very slowly towards each other, and typically no bipolar spindle assembly occurring in either first or second cycle (Movie S8). To test whether this phenotype may reflect a stronger centriolar defect, we performed immunofluorescence analysis and found that, indeed, ∼56% of first cell cycle embryos that exhibit a clear phenotype do not have any MTOC nor do they harbor a focus of centriolar or PCM signal (Fig. S6D–K). We confirmed that the RNAi phenotype in the first cell cycle is of paternal origin, since mating control males with hermaphrodites subjected to *sas-1 RNAi* rescues bipolar spindle assembly (Fig. 4F), but not viability (Fig. 4G), as for *sas-1* mutant animals (see Fig. 3I). Taken together, these results reinforce the notion that *sas-1* is needed for centriole integrity.

We raised antibodies against SAS-1, and found them to colocalize with the centriolar protein IFA (Fig. 4H), indicating that SAS-1 is a centriolar component. We found also that the centriolar signal is diminished in the progeny of animals injected with dsRNA directed against *sas-1*, attesting to the specificity of the antibodies (Fig. S6B–C). Intriguingly, in addition, we found that the signal detected by these antibodies is often present primarily on just one of the two centrioles in each spindle pole during anaphase of the first mitotic division following their disengagement (see Fig. 4H; 11/22 embryos). Centriolar localization was confirmed using GFP antibodies to label embryos expressing GFP-SAS-1 (Fig. 4I). We also determined that SAS-1 localizes to centrioles in sperm cells (Fig. 4K). Moreover, we found that SAS-1 distribution is not altered in *sas-1* mutant embryos (Fig. 4J), but that a fraction of centrioles in *sas-1* mutant and *sas-1(RNAi)* sperm cells lacks detectable SAS-1 signal (Fig. 4L–O), in line with the fully penetrant phenotype that they exhibit shortly after fertilization.

### SAS-1 is a stable component of centrioles recruited seemingly independently of other centriolar components

**Figure 5:**
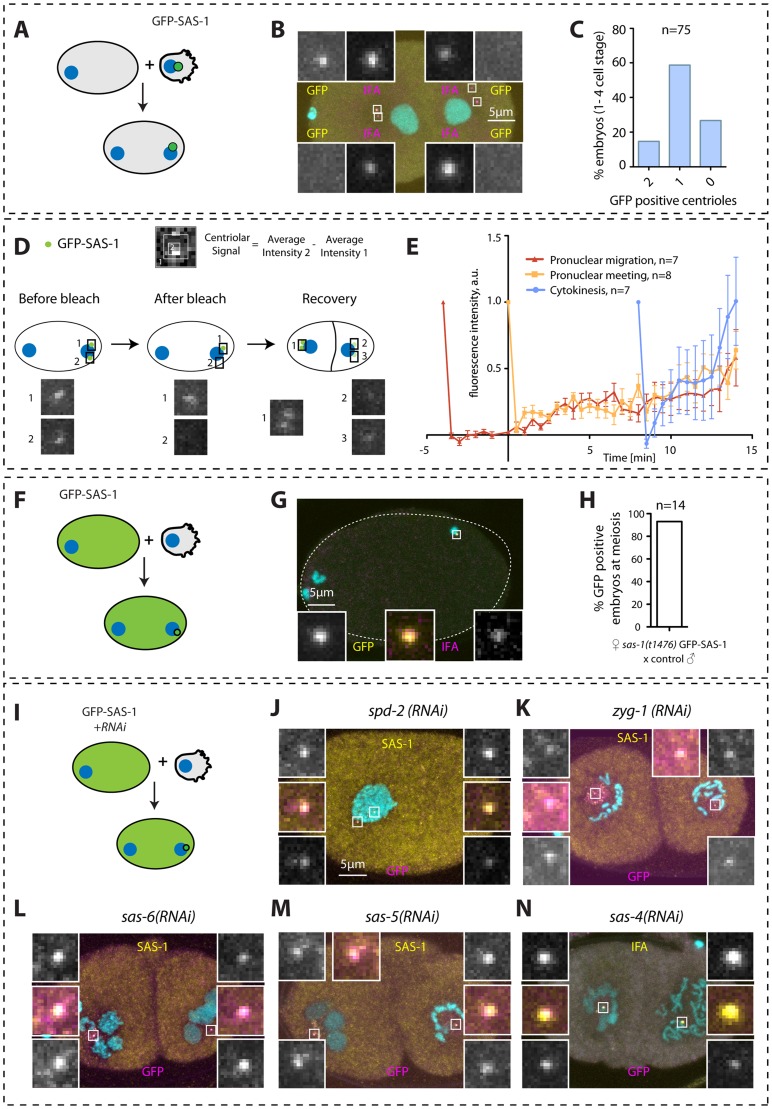
SAS-1 is a stable centriolar component recruited very early after fertilization independently of other centriolar proteins. (A, D, F, I) Schematics of experiments performed in the corresponding figures. (B, G) Embryos stained for GFP to reveal GFP-SAS-1 (yellow) and IFA (magenta). DNA is in cyan. Note that a GFP focus was not detected in some embryos, perhaps due to a very weak signal. However, some embryos exhibit just one very bright signal, supporting a *bona fide* asymmetry of SAS-1 distribution between the two sperm centrioles. Note that the embryo in B is in telophase of the one-cell stage. (E) Quantification of FRAP experiments performed with *sas-1(t1476)* GFP-SAS-1 embryos bleached at indicated time points. Centriolar signal intensity was quantified as depicted in (D). A schematic of an experiment performed at pronuclear meeting is shown in (D). (C, H) Quantification of experiments shown in B and G. Note that in C, embryos from the 1- until the 4-cell stage were scored. (J–N) Indicated components were inactivated using RNAi in GFP-SAS-1 hermaphrodites, which were mated with control males that contributed unlabeled paternal centrioles. Embryos were stained for SAS-1 (yellow) and GFP (magenta), except (N), where IFA was used instead of SAS-1 (yellow). DNA is in cyan. N = 10 for each condition.

Next, we tested whether SAS-1 is a stable component of centrioles by monitoring the fate of paternal centrioles carrying GFP-SAS-1 after fertilization. We found that paternally contributed GFP-SAS-1 is present in one focus in most embryos examined up to the four-cell stage (Fig. 5A–C). These findings establish that SAS-1 is a stable component of centrioles and suggests that the two paternally contribute centrioles may harbor different levels of the protein.

We were interested in addressing when during centriole biogenesis SAS-1 is recruited to the forming organelle. Thus, we performed fluorescent recovery after photobleaching (FRAP) experiments in embryos in the first cell cycle to address when SAS-1 is recruited onto centrioles. These experiments established that, irrespective of whether centriolar GFP-SAS-1 is bleached at pronuclear migration, at pronuclear meeting or just after cytokinesis, signal recovery is gradual thereafter, with a maximal pace of recruitment after cytokinesis (Fig. 5D–E). These findings lend support to the view that SAS-1 is recruited gradually onto the forming organelle and is stably associated with centrioles thereafter.

Next, we addressed how early after fertilization GFP-SAS-1 is recruited onto centrioles. To this end, we crossed control males with GFP-SAS-1 hermaphrodites and found that most centrioles are GFP-positive already shortly after fertilization (Fig. 5F–H). This result could be interpreted in two ways. First, SAS-1 may be a very early component recruited at the onset of centriole formation. Second, SAS-1 could be a very late component that associates with centrioles that are fully assembled. We favor the second possibility because of the gradual recovery following FRAP and because immunofluorescence analysis shows that some centrioles that are positive for IFA are negative for SAS-1 (Fig. 4H). The latter result suggests that SAS-1 has not yet been recruited to this subset of centrioles and hence is unlikely to be an early component. Furthermore, if SAS-1 were a component required for the initiation of procentriole formation, monopolar spindle formation would be expected in the second cell cycle upon maternal depletion, which is usually not the case (see also Discussion).

Next, we tested whether SAS-1 recruitment to centrioles shortly after fertilization depends upon other centriolar components. To this end, we depleted each of the five core centriolar components from GFP-SAS-1 hermaphrodites and mated them with control males. As shown in Fig. 5I–N, these experiments established that GFP-SAS-1 can localize to centrioles in embryos depleted of SPD-2, ZYG-1, SAS-6 or SAS-5, in which central tube formation is lacking, or depleted of SAS-4, in which the subsequent step of microtubule addition does not occur. Note that SAS-6, SAS-5 and SAS-4 remain present on sperm centrioles in these experiments, such that it is formally possible that SAS-1 recruitment shortly after fertilization depends upon the presence of these proteins on paternal centrioles.

In summary, we conclude that SAS-1 is a stable centriolar protein that tends to exhibit an asymmetric distribution on centriole pairs, and that can associate with existing centrioles without the need for the presence in the embryo of the five core centriolar components.

### SAS-1 binds to and stabilizes microtubules in human cells

**Figure 6:**
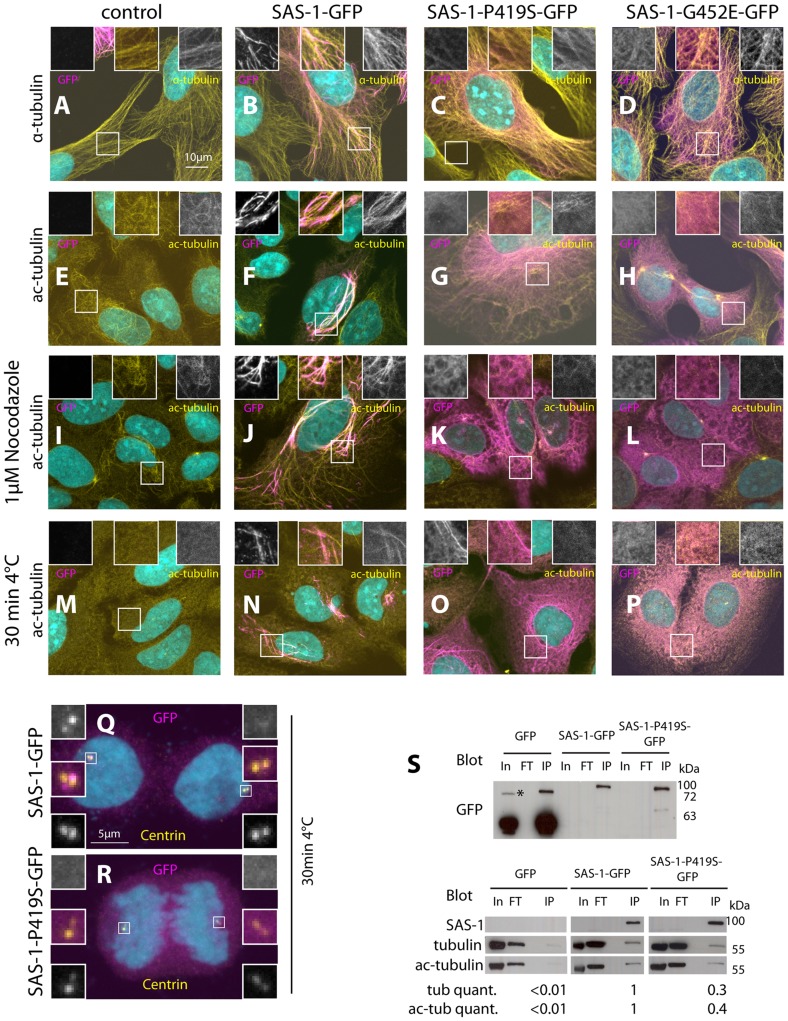
Overexpression of SAS-1 in human cells reveals microtubule binding and stabilization activities. (A–R) U2OS cells not expressing (control, A, E, I, M) or expressing wild type SAS-1-GFP (B, F, J, N, Q), SAS-1-P419S GFP (C, G, K, O, R) or SAS-1-G452E GFP (D, H, L, P) stained for GFP (magenta), as well as α-tubulin (A–D), acetylated tubulin (E–P) or centrin (Q–R) (yellow). DNA is in cyan. Insets in (A–P) are 2 fold magnified views, those in (Q–R) 3 fold magnified views. In (M–R), microtubules were depolymerized by placing cells 30 min on ice cold lead blocks, in (I–L), by treating them with 1 µM nocodazole for 1 h. (S) Immunoprecipitation using the GFP nanotrap of the indicated cell lysates probed with the indicated antibodies. In  =  Input  = 140 µg, FT  =  flow through  = 140 µg, IP  =  immunoprecipitation  = 20% of input. We used 2 mg of protein lysate for GFP, 10 mg for SAS-1-P419S-GFP, and 22 mg for SAS-1-GFP to obtain sufficient material. The tubulin and acetylated tubulin intensity was normalized with the SAS-1 band in SAS-1-GFP and SAS-1-P419S-GFP, and with the GFP band for GFP. Note that the upper blot is from a different membrane than the lower one. The asterisk indicates a probably dimeric form of GFP. N = 3 for the wild type and the mutant; representative blots are shown.

We set out to investigate the mechanisms by which SAS-1 stabilizes centrioles. Because proteins cannot be readily overexpressed in *C. elegans* embryos, we overexpressed SAS-1 in human cells instead. Intriguingly, we found that SAS-1-GFP colocalizes with microtubules in U2OS cells (Fig. 6A–B). Moreover, we noticed that in some cells, SAS-1-GFP is found adjacent to the centriolar protein Centrin, suggesting that SAS-1 can localize to centrioles also in human cells (Fig. 6Q). Furthermore, we addressed whether SAS-1 can associate with stable microtubules by staining cells for acetylated tubulin, a hallmark of such microtubules. Strikingly, we found that SAS-1-GFP exhibits extensive colocalization with acetylated microtubules (Fig. 6E–F). Together, these results indicate that SAS-1 can recognize stable microtubule configurations in this heterologous system, and raises the possibility that it could likewise associate with centriolar microtubules, which are also extremely stable.

We noted that cells expressing high amounts of SAS-1-GFP typically harbor more and thicker microtubule bundles (Fig. 6A–B). This raises the possibility that SAS-1 not only associates with stable microtubules but perhaps also promotes their stability. To address this possibility, we depolymerized microtubules using cold shock or nocodazole and found that under these conditions cells expressing SAS-1-GFP harbor more stable microtubules marked by acetylated tubulin than control cells (Fig. 6I–J, M–N). The conclusions reached using immunofluorescence analysis were confirmed in co-immunoprecipitation experiments, whereby SAS-1-GFP immunoprecipitates α-tubulin as well as acetylated tubulin (Fig. 6S). Taken together, these results establish that SAS-1 can bind and stabilize microtubules in human cells.

We next addressed whether the mutant versions of SAS-1 are impaired in their binding or stabilization activities in human cells. Importantly, we found that although the P419S and G452E mutant versions of SAS-1 localize to microtubules to some extent, they do not result in the generation of the numerous thick microtubule bundles observed with the wild type protein (Fig. 6C–D). In addition, the mutant proteins localize less extensively with acetylated tubulin (Fig. 6G–H), and SAS-1 P419S fails to localize to centrioles (Fig. 6R). Furthermore, we found that the mutant versions are not able to protect microtubules against cold- or nocodazole-induced depolymerization (Fig. 6K–L, O–P). Together, these observations suggest that the C2 domain is important for the microtubule binding and stabilization activities of SAS-1. This conclusion is supported by co-immunoprecipitation experiments, which revealed decreased association with α-tubulin and acetylated tubulin for the P419S mutant version of SAS-1 (Fig. 6S).

Taken together, these results indicate that SAS-1 binds and stabilizes microtubules in human cells. By extension, we propose that SAS-1 does so with centriolar microtubules in *C. elegans* (see Discussion).

### C2CD3 as a potential SAS-1 homolog in human cells

**Figure 7:**
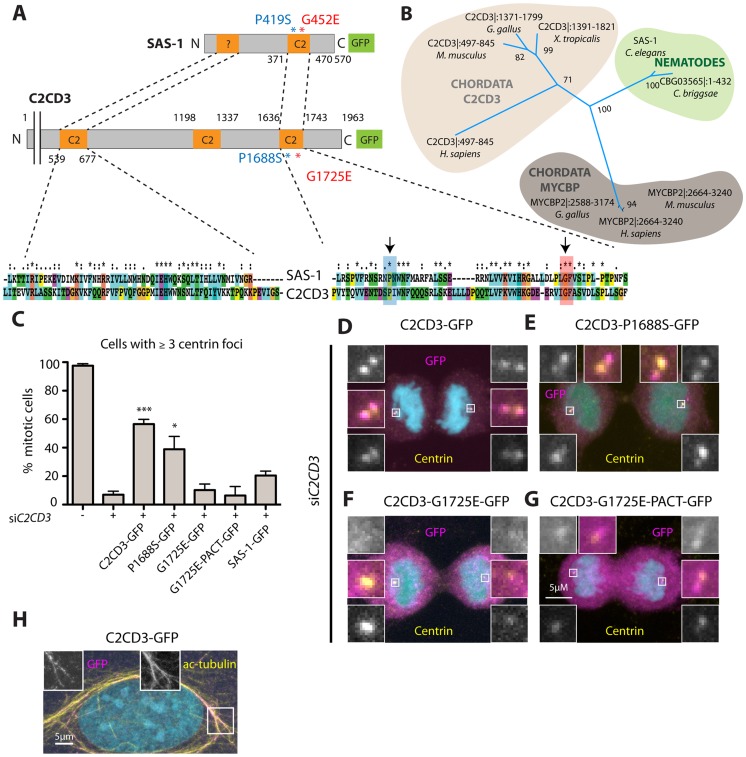
The human SAS-1 homolog C2CD3 is impaired by mutations important for SAS-1 function. (A) Schematics and alignment of the most homologous regions of *C. elegans* SAS-1 and human C2CD3. The arrows indicate the two *sas-1* mutations. Asterisks in the alignment indicate identity, colons strongly similar properties, dots weakly similar properties. Among the two proteins, the N-terminal C2 domain (C2CD3) and related region (SAS-1) share ∼30% identity, the C-terminal C2 domains ∼33% identity. (B) Phylogenetic tree based on PSI-BLAST analysis performed with full length SAS-1. The numbers indicate support values for the respective tree branches. Only selected species are shown; note that although widely present across metazoan evolution, a homolog was not identified in *Drosophila*. Note that we also identified a homology with MYCBP. However, when performing PSI-BLAST analysis with the N-terminus only, MYCBP did not come up, in contrast to C2CD3. (C) U2OS cells expressing the indicated fusion proteins were subjected to si*C2CD3*, stained for Centrin and GFP; the number of Centrin foci in the resulting mitotic cells expressing GFP were scored. Data from ≥3 experiments;>25 cells were counted in each condition, except for two experiments with G1725E PACT, where only very few cells (7 and 4, respectively) were positive for GFP at centrioles. Error bars indicate SEM, *** p<0.001, * p<0.05. (D–G) Mitotic cells depleted of C2CD3 and expressing C2CD3-GFP (D) or the indicated proteins harboring single amino acid substitutions corresponding to the *sas-1* mutant alleles (E–G) stained for GFP (magenta), centrin (yellow). DNA is in cyan. (H) An interphase cell expressing C2CD3-GFP showing microtubule co-localization of GFP (magenta) and acetylated tubulin (yellow).

We set out to address whether SAS-1 is evolutionarily conserved. BLAST analysis indicates that SAS-1 is well conserved amongst nematodes, and that proteins related to SAS-1 can be identified outside the nematode phylum. These include the potential SAS-1 human homolog C2CD3 (Fig. 7A–B). C2CD3 exhibits highest homology within the C2 domain of SAS-1, including conservation of the residues altered in the *sas-1* mutant alleles (Fig. 7A). Aligning SAS-1 and C2CD3 enabled us to identify a second domain in the N-terminal region of SAS-1 that bears resemblance to another C2 domain at the N-terminus of C2CD3 (Fig. 7A). Interestingly, C2CD3 is important for the assembly and/or maintenance of distal elements of centrioles in human and mouse cells [44]–[46]. Moreover, C2CD3 is important for primary cilium formation during mouse embryogenesis [47] and is mutated in an oral-facial-digital (OFD) syndrome [45].

Endogenous C2CD3 localizes notably to the distal end of human centrioles [44], and we found in addition here that C2CD3-GFP colocalizes with acetylated microtubules in some cells, reminiscent of the observations with SAS-1-GFP. Such colocalization has also been observed when overexpressing mouse C2cd3 in human cells [45]. Although we found that SAS-1-GFP cannot rescue C2CD3 depletion in human cells (Fig. 7C), we wanted to test if mutations analogous to those that impair SAS-1 function in *C. elegans* also impact C2CD3 function in human cells. To this end, we expressed the two corresponding mutant versions of C2CD3, P1688S and G1725E, and analyzed their capacity to rescue depletion of endogenous C2CD3. We found that P1688S can rescue C2CD3 depletion to some extent, while G1725E cannot (Fig. 7C), in line with *sas-1(t1521)* being a more severe allele than *sas-1(t1476)* in *C. elegans* (see Table S1). Moreover, we found that whereas wild type C2CD3-GFP localizes to centrioles, G1725E does not (Fig. 7D, F). In an attempt to test if this lack of centriolar localization is responsible for the lack of function, we fused the G1725E mutant to a PACT domain to target it to centrosomes [48]. We found that whereas the PACT domain directs the protein to centrosomes in some cells (Fig. 7G), there is no rescue of the phenotype incurred upon C2CD3 depletion (Fig 7C). This may be because the PACT domain targets the protein to the incorrect centrosomal location or else reflect the fact that the G1725E mutation impairs activity in addition to centriolar targeting. Regardless, these results together suggest that C2CD3 may be a *bona fide* SAS-1 functional homolog in human cells.

## Discussion

Our findings identify SAS-1 as a novel protein critical for centriole integrity and, thereby, for proper centriole formation. SAS-1 is a C2 domain containing protein that stably associates with centrioles, and that can also bind and stabilize microtubules, leading us to propose that this is the mechanism by which it acts to ensure centriole integrity.

### Dual parental requirement for *sas-1*

Our analysis uncovered both a paternal and a maternal requirement for *sas-1* function (Fig. S7). When paternal function is lacking, centrioles contributed by *sas-1* mutant sperm loose their integrity following fertilization, and a monopolar spindle assembles in the first cell cycle. When maternal function is lacking, centrioles loose their integrity with a given probability later during embryogenesis, leading to cell division defects usually from the third cell cycle onwards. In contrast, essentially all centrioles contributed by *sas-1* mutant sperm are affected, indicating that SAS-1 plays a particularly important role during spermatogenesis to ensure the maintenance of paternally contributed centrioles after fertilization. Perhaps the centrioles made during male gametogenesis are particularly sensitive because of their extreme persistence compared to those made in rapidly proliferating cells. Alternatively, SAS-1 may protect centrioles from the mechanism that eliminates centrioles during oogenesis, which may conceivably remain somewhat active after fertilization.

The phenotypes exhibited by embryos lacking either paternal or maternal SAS-1 function support the notion that SAS-1 is recruited to centrioles after centriole assembly has occurred. Indeed, if SAS-1 were required early during centriole biogenesis, a failure in centriole formation would be expected already during the meiotic divisions of spermatogenesis, leaving mature sperm with just one centriole, as in *zyg-1* or *sas-5* mutant animals [5], [6]. Instead, our analysis demonstrates that there are two paternally contributed centrioles in *sas-1* mutant sperm, which loose integrity following fertilization. Similarly, a requirement early during centriole biogenesis would be expected to give rise to monopolar spindle assembly in the second cell cycle upon depletion of the maternal contribution of SAS-1, as upon that of *zyg-1*, *sas-5*, *sas-6* or *sas-4* [5]–[10]. Instead, the phenotype upon maternal depletion of *sas-1* becomes apparent only in later cell cycles, as expected from a requirement to stabilize centrioles generated in the embryo. We note, however, that SAS-1 function seems to be required for the generation of structurally normal centrioles, as evidenced by the absence of centriolar microtubules visible by ultrastructural analysis of *sas-1* mutant embryos during the first cell cycle (Fig. 2Q–R). Overall, we conclude that SAS-1 is a component that acts to ensure the integrity of centrioles both during their assembly and thereafter.

### The impact of SAS-1 on SAS-6

Our work established that SAS-1 stabilizes paternally contributed centriolar SAS-6 shortly after fertilization, whereas the requirement for the stabilization of centriolar SAS-4 becomes apparent only later in the cell cycle. In principle, SAS-1 could exert a dual function, one in stabilizing microtubules and one in maintaining centriolar SAS-6. A similar dual function has been proposed for *Tetrahymena* Poc1, which localizes not only to centriolar microtubules, but also to the cartwheel region where SAS-6 proteins reside [33]. Intriguingly, in addition, a fraction of human cells depleted of Poc1A and Poc1B assemble tripolar spindles [35], reminiscent of the situation in *sas-1* homozygous mutant embryos and embryos maternally mutant for *sas-1* (see Fig. S7).

A simpler alternative to invoking a dual function for SAS-1 is that impaired centriolar microtubule stability could result in the loss of the central tube, and thus of its constituent protein SAS-6. Regardless of the actual mechanism, the focus of β-tubulin and SAS-4 that is left in the mutant condition likely reflects trapping of these proteins in the location of what used to be a centriole. In this context, it is interesting to note that SAS-4 can persist in a focus without SAS-6 (see Fig. 2), indicating that SAS-6 maintenance is not required for SAS-4 maintenance, in contrast to the relationship during centriole biogenesis [11], [12]. Importantly, these observations also demonstrate that, similarly to what is observed in human cells where HsSAS-6 is absent from the parental centriole [49], SAS-6 is not essential on the old centriole to foster the formation of a new centriole-related structure in *C. elegans*.

### Unequal *C. elegans* sperm centrioles

Analyzing the fate of the two centrioles contributed by *sas-1* mutant sperm revealed that one of them retains integrity for a longer time than the other (see also Fig. S7), suggesting that the two sperm centrioles are not identical. Perhaps the age difference between the two sperm centrioles, one having been formed between meiosis I and meiosis II, and the other one being older, is what determines sensitivity to *sas-1* impairment. Differential stability of centrioles has also been proposed in human cells based on the observation that single centrioles were observed in some cells upon injection with polyglutamylated antibodies [31], [50]. The differential requirement of the two sperm centrioles for SAS-1 function ties with the observation that GFP-SAS-1 is most often detected on only one of the two paternally contributed centrioles (see Fig. 5A–C), perhaps the one that relies most on its function.

The two sperm centrioles are not identical in most metazoan organisms, including the majority of vertebrate species. For instance, in human sperm, one centriole seeds the formation of the axoneme of the flagellum and is degenerate, while the other centriole seems to remain intact [51]–[53]. By contrast, in *C. elegans*, in which sperm cells are amoeboid, ultrastructural analysis failed to reveal any difference between the two centrioles [5]. The unequal distribution of GFP-SAS-1 may provide the first molecular means to distinguish between these two entities.

### SAS-1 can bind and stabilize microtubules

We showed that SAS-1 can bind and stabilize microtubules in human cells. Since SAS-1 mutant proteins do not bind or stabilize microtubules as efficiently, the C2 domain must be important for these activities. How do findings in such a heterologous system relate to SAS-1 function in *C. elegans*? Centriolar microtubules are not known to be post-translationally modified in *C. elegans*; in fact, the α-tubulin isoforms expressed in the early embryo do not have the K40 residue required for their acetylation [54]. Nevertheless, centriolar microtubules are also stable in *C. elegans* embryos, as evidenced by their resistance to cold treatment [55]. One possibility is that SAS-1 recognizes a conformation characteristic of stable microtubules, rather than a specific modification on α- or β-tubulin, and that such a conformation characterizes both stable cytoplasmic microtubules in human cells and centriolar microtubules in both human cells and *C. elegans* embryos. Another, more radical, hypothesis is that in the absence of tubulin post-translational modifications in the early *C. elegans* embryo, there must be another system to stabilize centriolar microtubules. SAS-1 could be part of such a hypothetical system and thus guarantee centriolar microtubule stability.

### SAS-1 as a platform towards studying related human protein

Bioinformatic analysis indicates that C2CD3 is a putative human homolog of SAS-1. C2CD3 also acts late, being needed for the presence of hPOC5 and Centrin in the distal part of centrioles [44]. Interestingly, upon hPOC5 depletion in human cells, centrioles are short and comprise only microtubule doublets instead of the usual triplets [56]. We speculate that human cells depleted of C2CD3 may have impaired centriolar microtubules and/or centriole integrity. In agreement with this suggestion, it has been reported that centrioles in fibroblasts of C2CD3 mutant mice are shorter and lack appendages [45], although another study reported no such defect [46]. Whether centrioles in such mutant C2CD3 cells harbor microtubule triplets is unclear [45].

Our findings with SAS-1 also have potential implications for human disease-causing genes. Suggestively, patients suffering from an oral-facial-digital (OFD) syndrome harbor a single amino acid substitution in a C2 domain of the SAS-1 homolog C2CD3 [45]. Moreover, the two *sas-1* mutations lie in evolutionarily conserved residues amongst C2 domains, together suggesting that C2 domains are important for function in this protein family. Intriguingly, the equivalent of the P419S mutation has been implicated in Joubert syndrome ciliopathy, with a P1122S alteration in the C2 domain containing protein CC2D2A [57]. Moreover, several proteins that localize to the transition zone between the centriole and the primary cilium contain C2 domains, including NPHP1/4/8. Mutations in the genes encoding these three proteins can lead to nephronophtisis, a cystic kidney ciliopathy. Hence, mutations in the residues we describe here may be uncovered in upcoming investigations of ciliopathies (reviewed in [58]).

In conclusion, we discovered SAS-1 as a novel C2 domain containing protein that can bind and stabilize microtubules. SAS-1 is required most critically during spermatogenesis to maintain centriole integrity in the newly fertilized embryo, and thus plays an essential role in ensuring that intact centrioles are passed on from one generation to the next.

## Materials and Methods

### Nematodes and RNAi

Nematode culture was according to standard procedures [59]. The parental *sas-1(t1476) unc-32(e189)/*qC1 *dpy-19(e1259) glp-1(q339)* and *sas-1(t1521) unc-32(e189)/*qC1 *dpy-19(e1259) glp-1(q339)* strains [42] were crossed to different strains expressing fluorescent fusion proteins to obtain suitable transgenic animals. When males were needed, we used *sas-1(t1476)* simply balanced by hT2. *fog-2(q71)* [60], *plg-1(e2001)* [61], CB4856 [62], CB4118 (*unc-32(e189)*, *ooc-4(e2078)*; eDf2, GFP-SAS-6 [63], GFP-SAS-4 [64], *rrrSi212[Psas-5::gfp:sas-5(synthetic)::sas-5 3′UTR; unc-119(+)] (II), unc-119(ed3) III; ijmSi8 [pJD362/pSW077; monII_5′mex-5_GFP::tbb-2; mCherry::his-11; cb-unc-119(+)]*I (a gift from Julien Dumont). mCherry-SAS-4 [65], mCherry-H2B was obtained by outcrossing OD57 (mCherry-H2B GFP-α-tubulin) [66] to N2 animals and segregating away GFP-α-tubulin. For generating worms expressing GFP-SAS-1, the cDNA of Y111B2A.24 was cloned in frame into pSU25, which expresses GFP under *pie-1* regulatory elements and carries also *unc-119(+)*. The GFP-SAS-1 fusion construct was bombarded into *unc-119 (ed3)* worms using a gene-gun (Biodrad) [67], and resulting animals with wild type locomotion were crossed into the *sas-1(t1476)* mutant background, resulting in GZ1006.

RNAi by feeding was performed according to standard procedures. L4 worms were subjected to *sas-1(RNAi)* for 96 h at 20°C and the F1 (when analyzing sperm) or F2 (when analyzing embryos) of these animals were analyzed; to *sas-5(RNAi)* for 24 h at 20°C; to *sas-6(RNAi)* for>20 h at 20°C; to *sas-4(RNAi), spd-2(RNAi)* and *zyg-1(RNAi)* for 48 h at 20°C.

To ensure that cross-progeny was examined, in some experiments we utilized otherwise wild type feminized *fog-2*(*q71*) hermaphrodites that do not produce sperm, or *plg-1*(*e2001*) males that leave a clearly visible mating plug in the vulva of the hermaphrodite, but that are otherwise wild type. For simplicity, we refer to *fog-2*(*q71*) hermaphrodites as “control hermaphrodites” and to *plg-1*(*e2001*) males as “control males” throughout the text.

*sas-1* dsRNA (nucleotides 15–1252) was generated by *in vitro* transcription (MEGAscript, Lifetechnologies) from the SP6 or T7 promoters followed by RNeasy (Qiagen) clean up and double stranding. dsRNA was injected into the gonads of young adult hermaphrodite that were then allowed to recover for ∼24 h at 20°C, after which analysis by immunofluorescence analysis or DIC time-lapse microscopy was conducted.

### Indirect immunofluorescence

To release embryos, worms were dissected in ∼5 µl dH_2_O on slides coated with 2 µg/µl poly-L-lysine in PBS, then fixed and stained as described for indirect immunofluorescence of centriolar proteins [6]. Briefly, embryos were fixed in ice-cold methanol for <2 min and blocked in 2% bovine serum albumin (BSA) for 15–20 min prior to incubation with primary antibodies overnight at 4°C. Human cells were fixed for 7 min in ice-cold methanol, washed in PBS and blocked for 1 h with 1% BSA 0.05% Tween-20 in PBS, followed by incubation with primary antibodies overnight at 4°C. The following primary antibodies raised in rabbits were used at the indicated concentrations: 1∶800 SAS-4 [8], 1∶50 SAS-5 [6], 1∶1000 SAS-6 [7], 1∶1000 ZYG-1 [8], 1∶1000 SPD-2 ([11], gift from Michael Glotzer), 1∶5000 SPD-5 ([68] gift from Bruce Bowerman), 1∶1000 GFP (gift from Viesturs Simanis). The following primary antibodies raised in mouse were utilized: 1∶500 α-tubulin (DM1α, Sigma), 1∶200 α-tubulin-FITC (Invitrogen), 1∶1000 acetylated tubulin (T6793; Sigma), 1∶50 IFA [69] and 1∶3000 centrin-2 (20H5; gift from Jeffrey L. Salisbury). Secondary antibodies were goat anti-mouse coupled to Alexa 488, goat anti-rabbit coupled to Alexa 568, donkey anti-rabbit coupled to Alexa 594, and goat anti-mouse coupled to Cy5, all used at 1∶500 for *C. elegans* and 1∶1000 for human cells (Molecular Probes). Slides were counterstained with ∼1 µg/ml Hoechst 33258 (Sigma) to visualize DNA.

### Mapping and sequencing of *sas-1* locus

For mapping, *unc-32(e189) sas-1(t1476)* or *unc-32(e189) sas-1(t1521)* hermaphrodites were crossed to CB4856 males. Unc-Emb and Unc-non-Emb F2 recombinants were recovered from the heterozygous F1s. SNP mapping [70] was then used to narrow down the locus to a 100 kbp region between SNPs WBVar00567469 and WBVar00182099. For sequencing, genomic DNA was extracted from>1000 homozygous *unc-32(e189) sas-1(t1476)* worms. Next generation Illumina Technology Sequencing (FASTERIS) revealed 5 SNPs and Indels in the region of interest. A C1255T nucleotide change was identified in the sequence of Y111B2A.24, translating into a P419S substitution in the C2 domain. A G1355A nucleotide change was found by Sanger sequencing in *unc-32(e189) sas-1(t1521)*, resulting in a G452E change in the C2 domain. Sanger sequencing of the third *sas-1* allele, *sas-1(t1538)* [42], showed that it harbors the same single nucleotide change as *sas-1(t1521)*. Hence, experiments conducted with *sas-1(t1538)* are not reported in the manuscript.

### SAS-1 bioinformatic analysis

The full-length amino acid sequence, the C2 domain and the N-terminal 220 amino acids of SAS-1 were each used to identify homologous sequences using BLAST and PSI-BLAST. Keeping an E-value of <10^−5^, after ∼7 PSI-BLAST iterations not more than 7 new proteins were detected for any of the queries. While we did not detect C2CD3 using the C2 domain as input, C2CD3 was the best hit when using both the N-ter and the full length. A single BLAST analysis of SAS-1 against the human proteome identified C2CD3 as the second best hit, with Rabphilin 3A being first due to higher similarity in the C2 domain. However, Rabphilin 3A has another closer homolog in *C. elegans* (RBF-1) and quickly disappears with PSI-BLAST iterations. Conversely, blasting human C2CD3 against the *C. elegans* proteome identified SAS-1 as the second hit, with C07A12.7 being first. This protein has another closer homolog in human cells, TOM-1. The multiple sequence alignment was calculated with MAFFT [71] and the phylogenetic tree generated using RAxML [72] and visualized in Dendroscope [73]. The following are the accession numbers of the proteins shown in Fig. 7B: *G. gallus* XP_004939073.1 (C2CD3) and XP_004938682.1 (MYCBP2), *X. tropicalis* NP_001072727.1, *M. musculus* NP_001273506.1 (C2CD3) and XP_006518463.1 (MYCBP2), *H. sapiens* NP_001273506.1 (C2CD3) and XP_005266356.1 (MYCBP2), *C. briggsae* XP_002647047.1. Molecular graphics and analyses were performed with the UCSF Chimera package, developed by the Resource for Biocomputing, Visualization, and Informatics at the University of California, San Francisco (supported by NIGMS P41-GM103311) [74].

### SAS-1 antibody

The N-terminal 441 nucleotides of the *sas-1* coding sequence were cloned C-terminally into pMGWA or pHGWA to express MBP-SAS-1N or His-SAS-1N, respectively, which were purified using standard procedures. MBP-SAS-1 was injected into rabbits according to standard protocols (Eurogentec). For affinity purification, His-SAS-1N was blotted on a membrane and the clarified serum incubated with the membrane. Antibodies were eluted using 0.1 M glycine at pH 2.3 and neutralized with 1.5 M Tris-HCl, pH 8.8. Antibodies were dialysed against PBS overnight at 4°C and stored at −80°C or at −20°C in 50% glycerol. SAS-1 antibodies were used at a dilution of 1∶50 or 1∶100.

### Microscopy and live imaging

Time-lapse differential interference contrast (DIC) microscopy of early embryos was performed as described [42]. Dual time-lapse and fluorescence DIC imaging was performed on a Zeiss Axioplan 2 [75]. The motorized filter wheel, two external shutters, and the 1,392×1,040 pixels 12-bit Photometrics CoolSNAP ES^2^ were controlled by µManager. Images were acquired with an exposure time of 10–100 ms for the DIC and 250 ms for the fluorescence channels using the Zeiss Filter Set 10 (GFP) and 400 ms for 43HE (mCherry). Indirect immunofluorescence was imaged on an LSM700 Zeiss confocal microscope, using 0.5 µm–1 µm optical slices. Images were processed using ImageJ.

### Human cells

U2OS cells were cultured at 37°C and 5% CO_2_ in DMEM supplemented with GlutaMAX (Invitrogen, Carlsbad, CA) and 10% fetal calf serum. Inducible cell lines were generated using a tet-on system [76]. Briefly, cells grown to ∼80% confluency in a 10 cm dish were transfected with ∼7 µg DNA and after 4–6 h the medium was changed. The next day, cells were exposed to medium containing 1 µg/ml puromycin and selected for 1–2 weeks. In experiments with C2CD3-GFP, we used isoform 2 of the protein (XP_056346.3), and it should be noted that there is also a longer isoform 1 (XP_001273506.1). For depletion of C2CD3, cells were transfected with 20 nM si*C2CD3* at 0 h as well as 48 h, and cells fixed at 96 h. For rescue experiments, doxycylin was typically added 24 h after the initial siRNA transfection.

To depolymerize microtubules, cells grown in 6-well plates were incubated with 1 µM nocodazole for 1 h or 30 min on lead blocks placed on ice and fixed directly after washing in cold PBS with ice cold methanol. Cells were then washed and incubated with antibodies as described above.

Immunoprecipitation experiments were performed using GFP-nanotrap (Chromotek). Briefly, cells were collected following trypsinization, washed with PBS, lysed in MT-buffer (50 mM Tris-HCl, pH 7.5, 0.5 mM EDTA, 0.5% NP40, 150 mM NaCl) and incubated with ∼30 µl beads overnight. The following day, beads were washed 3× with MT-buffer and sample buffer was added to the beads. Quantification of western blots was performed in Fiji, using the GelAnalyzer toolkit.

### Transmission electron microscopy

For *C. elegans* embryos, the samples were cryo-immobilized using an EMPACT2+RTS high-pressure freezer (Leica Microsystems), and freeze-substituted at −90°C for 20 h in acetone containing 1% osmium tetroxide and 0.1% uranyl acetate. The temperature was raised progressively to room temperature over 22 h in an automatic freeze-substitution machine (Leica EM AFS) and samples were thin-layer embedded in Epon/Araldite as published [12]. Thin sections (70 nm) were cut using a Leica Ultracut UCT microtome and collected on Formvar-coated copper grids. Sections were post-stained with 2% uranyl acetate in 70% methanol followed by aqueous lead citrate and viewed in a TECNAI 12 (FEI) transmission electron microscope operated at 80 kV.

For sperm analysis, worms were preserved for transmission electron microscopy using high pressure freezing and freeze substitution. Briefly, worms were loaded in the 3 mm aluminum carrier along with *E. coli* food paste and frozen with a high-pressure freezer (Leica HPM100, Leica Microsystems). The samples were then placed into tubes held in liquid nitrogen and containing frozen acetone with 1% osmium tetroxide, 0.5% uranyl acetate, 5% water. The tubes were then warmed inside an ice bucket containing dry ice, and left overnight agitating gently on a shaker table. The following day, the dry ice was removed and the ice bucket and tubes allowed to warm to 0°C over the next 2 hours. The samples were then washed three times with fresh acetone at 5°C and then embedded in a graded series of epon resin-acetone mix, until pure resin. Once completely infiltrated, the samples were arranged in silicon molds and the resin cured for 48 hours at 65°C.

Serial sections of the gonad region were cut using a diamond knife and ultramicrotome (Leica UC7). Sections were collected on single slot copper grids with a formvar support film, stained with lead citrate and uranyl acetate and imaged inside a transmission electron microscope at 80 kV (Tecnai Spirit, FEI Company), using a CCD camera (Eagle, FEI Company).

### Fluorescent Recovery after Photobleaching (FRAP) and live imaging

*sas-1(t1476)* mutant embryos expressing GFP-SAS-1 were filmed on a LSM700 Zeiss confocal with a 40× objective, acquiring 10–20 µm optical sections at each time point with a time frame of 30 s at a resolution of 1024×1024 pixels. A square of varying size was drawn around a centrosome and bleached with 50 iterations and 100% laser power. For quantification, a square of 10×10 pixels was drawn around the centriole. The outer 64 pixels were measured for background subtraction while in the inner region of the 36 remaining pixels, the brightest 3×3 pixels were quantified as the centriolar signal. Then, the mean pixel intensity of the outer background region was subtracted from the bright inner region.

For tracking of tripolar divisions, embryos were imaged on a Yokogawa Spinning Disk Scanning Unit, using a 63× lens and 20 s frame rate over a time of 20–50 min.

### Modeling of *sas-1* maternal contribution

We define the probability of a sequence of cellular divisions *D_i_* given a parameter set termed *θ* (i.e. *{P_D1_, P_D2_}*) as:
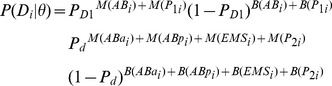
where *P_D1_* is the probability of a C1 centriole to disintegrate right away, and *P_D2_* the probability of a C1 centriole to disintegrate after one cell cycle, *P_d_* that of a daughter centriole to disintegrate (i.e. either after one or two cell cycles): 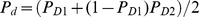



*θ* is either (*P_C1_* = *P_C2_*) in the simple model 1 or (*P_C1_*, *P_C2_*) in model 2 and *M(x)* and *B(x)* are functions defining the type of division cell *x* has undergone):
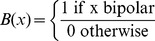


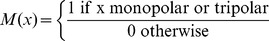
thus divisions that could not be assessed visually are ignored (i.e. both *B(x)* and *M(x)* are 0). Using this framework, we define the probability of the division dataset *D* given a parameter set *θ* as:
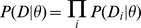



To obtain the marginal posterior probability distributions displayed in Fig. 3, we sampled *P(D|θ)* using a custom Matlab implementation of the Delayed Rejection Adapted Metropolis (DRAM, [77]) algorithm, based on their published code.

The p-value computed to determine which model to favor was based on a maximum log-likelihood ratio test.

## Supporting Information

Figure S1 *sas-1* mutant embryos harbor only one MTOC at PNM. Wild type (A–B, E–F, I–J, M–N, Q–R, U–V) or *sas-1(t1476)* (C–D, G–H, K–L, O–P, S–T, W–X) embryos were stained for α-tubulin (cyan), IFA (yellow) and SPD-5 (A–D), SPD-2 (E–H), ZYG-1 (I–L), SAS-6 (M–P), SAS-5 (Q–T) or SAS-4 (U–X) (all magenta). DNA is shown in red. Note that only one MTOC in (C) is IFA positive. Note also that in (W) two IFA foci are visible, but only one harbors SAS-4; the second does not harbor SAS-4 nor is it an MTOC, suggesting that this is a degenerate centriole Note finally that both MTOCs in (X) do not harbor any centriolar marker. See also Table S2.
(TIFF)

Figure S2 Monitoring the root of tripolar spindle assemblies in *sas-1* embryos. *sas-1(t1476)* homozygous animals expressing GFP-SAS-6 and mCherry-β-tubulin were imaged on a Spinning Disk microscope. (A) Individual MTOCs and corresponding GFP-SAS-6 foci from the embryo in (B) are shown. (B) A representative embryo is shown. After monopolar spindle assembly in the first cycle, a tripolar spindle forms in the second cycle. Note that the embryo harbors two GFP-SAS-6 foci and two MTOCs during pronuclear migration, but looses one before pronuclear meeting. Note also that after the first monopolar mitosis, one MTOC forms without an initial GFP-SAS-6 focus and seems to split off from the already existing MTOC (indicated by white arrow); another MTOC looses GFP-SAS-6. Note that this is in line with the immunofluorescence analysis (Fig. S1C), where some embryos were found to harbor two MTOCs of different sizes. Time is in min and sec, with 00:00 corresponding to pronuclear meeting.
(TIFF)

Figure S3 *sas-1(t1476)* sperm cells have no apparent centriolar defect. (A–B) Electron micrographs of serially sectioned wild type (A) or *sas-1(t1476)* (B) sperm cells. Centrioles are indicated with arrows. The microtubule blades are schematized in (A′) and (B′). Shown are single 60 nm sections. Due to the difficulties of conducting EM with the minute *C. elegans* centrioles in sperm cells, one cannot conclude with utmost certainty whether centriole ultrastructure is fully intact in the mutant. Note also that apparent abnormalities were observed occasionally in the perinuclear ring of *sas-1(t1476)* sperm cells, which was absent or less visible than in the wild type (arrowheads). Furthermore, we often observed extraneous densities elsewhere in the mutant cells (not visible here). 12 wild type and 8 *sas-1(t1476)* sperm cells coming from one animal each were analyzed by serial sectioning. n  =  nucleus, arrows indicate centrioles. (C–H) Wild type (C, E, G) or *sas-1(t1476)* (D, F, H) sperm cells stained for SP-56 (green) to label sperm membranous organelles and with SAS-6 (C–D), SAS-5 (E–F) or SAS-4 (G–H) (red in merge and alone in magnified insets). DNA is shown in blue. (I) Quantification of experiments shown in (C–H).
(TIFF)

Figure S4 Distribution of paternally contributed SAS-4 and SAS-6 in *sas-1* mutant embryos. (A) Quantification of embryos shortly after fertilization stained with SAS-6. (B) Quantification of embryos shortly after fertilization stained with SAS-4 (experiments shown in C–D). Centrioles are often too close to be observed as two separate entities in these early stages. (C–D) Immunostainings of a wild type (C) or *sas-1(t1476)* (D) embryo for α-tubulin (green), SAS-4 (red), IFA (grey). DNA is shown in blue. Note the two disengaged centrioles in (D).
(TIFF)

Figure S5 Recruitment of maternal centriolar components is not affected in *sas-1* mutant embryos. (A, G) Schematic of experiments performed in (B–E) and (H–I). (B–E) Control (B, D) or *sas-1(t1476)* (C, E) males expressing mCherry SAS-4 mated to control animals expressing either GFP-SAS-4 (B–C) or GFP-SAS-6 (D–E). Stills of the GFP channel from time-lapse movies are shown; for simplicity, the mCherry signal is not shown. However, we noted that in some *sas-1* embryos, the paternal mCherry signal could no longer be detected, in line with the data reported in Fig. 2. (F) Quantification of experiments performed in (B–E). (H–I) Control (H) and *sas-1(t1476)* (I) males expressing mCherry-H2B were mated to *sas-1(t1476)* animals expressing GFP-SAS-6. Only animals with mCherry positive paternal DNA were analyzed, since this time we did not mate males to feminized control animals but instead to hermaphrodites. Stills from time-lapse movies are shown. For simplicity, the mCherry signal is not shown. (J) Quantification of experiments performed in (H–I).
(TIFF)

Figure S6 *sas-1(RNAi)* results in centriole loss and GFP-SAS-1 can rescue the *sas-1(t1476)* mutant phenotype. (A) Progeny test of indicated conditions, all performed at 24°C. (B–C) Four-cell stage wild type (B) embryos and embryos following injection with *sas-1* dsRNA (C) stained for α-tubulin (cyan), SAS-1 (magenta and shown alone in insets) and IFA (yellow). DNA is shown in red. Arrows indicate tripolar spindles; dashed lines indicate the cell from which the insets originate. The vast majority of embryos derived from such injected animals exhibited multipolar spindle assembly in at least one blastomere at the 4-cell stage and thereafter. In the wild type, 81% (n = 43) centrioles were strongly SAS-1 positive and none were SAS-1 negative. In embryos from *sas-1* dsRNA injected animals, only 27% (n = 104) were strongly SAS-1 positive (a number that includes the paternally contributed and RNAi resistant sperm centrioles) whereas 58% were SAS-1 negative. Note that the cytoplasmic signal seems to be unspecific, since it is not diminished -but rather increased- in *sas-1(RNAi)* animals. (D–K) Immunostainings of wild type (D–E, H–I) or *sas-1(RNAi)* embryos (F–G, J–K) for α-tubulin (green), IFA (grey) and SPD-5 (D–G) or SAS-6 (H–K) (red). DNA is shown in blue. Note the absence of a clear microtubule network in *sas-1(RNAi)* embryos. N = 11 embryos.
(TIFF)

Figure S7 Working model of SAS-1 function and phenotypic consequences of its absence. Simplified centrioles are shown, with SAS-6 (green) building the central tube and SAS-4 (red) the remainder of the structure. Microtubules are shown as grey cylinders or, if abnormal, as wireframe cylinders or cylindrical fragments. C0, centrioles in sperm; C1 centrioles formed next to C0s; C2s centrioles formed next to C1s. Wild type (A–E), *sas-1* homozygous animals (F–L), *sas-1* mutant sperm fertilizing wild type oocytes (M–Q), wild type centrioles fertilizing *sas-1* mutant oocytes (R–W). The different stages are indicated with 1–7. Note that in (G) and (N), two centrioles are still present as judged by SAS-4, but one of them will disappear shortly thereafter. Note that in (I), the C1 centriole has an abnormal microtubule organization that is not recognized as a normal centriole by electron microscopy. After (I), three types of phenotypes can be observed, monopolar (J), bipolar (K) or tripolar (L) spindle assembly, which we explain as follows. For monopolar spindle assembly (J), we surmise that C0 disappeared after fostering formation of C1. Bipolar spindle assembly (K) follows from C0 still being sufficiently present to foster a C1, and the C1 made in the first cycle now fostering a C2; this results in a bipolar spindle, with each pole harboring at least one centriole or centriole-like structure. For tripolar spindle assembly (L), we surmise that C0 breaks apart during mitosis, giving rise to a tripolar configuration. In (N), wild type maternally provided SAS-1 can stabilize centrioles derived from *sas-1* mutant sperm to some extent, giving rise to some rescue (see Fig. 1E). In (P), the C1 forms in the presence of wild type maternally provided SAS-1, which presumably leads to formation of a normal procentriole. Accordingly, all divisions thereafter are bipolar (Q). In (V), we hypothesize that the C1s made in (T–U) cannot be stabilized, but can still give rise to C2s (similar to the case in (H) or (O)). Consequently, we hypothesize that whereas the C0s are intact in (W), the C1s are unstable and thus disintegrate. This situation is reminiscent of the scenario in (J–L); accordingly, we also observe cases of monopolar and tripolar configurations. Note that in (W), only the situation before centriole duplication is shown for simplicity.
(PDF)

Table S1 Phenotypes of *sas-1* mutant embryos at 24°C. (A–B) Analysis of indicated embryos by time-lapse DIC microscopy in the first cell cycle (A) and in the second cell cycle for the subset of embryos that exhibited monopolar spindle assembly in the first cell cycle (B). (C) Embryonic viability amongst the progeny of animals of the indicated genotypes. We used feminized *fog-2(q71)* animals that do not produce sperm to rigorously test the paternal contribution of *sas-1(t1476)*. ° Although we dissected 21 *sas-1(t1476)*/*eDf2* animals, we could score the first cell divisions in only six of the resulting embryos, because these animals were either sterile (n = 5) or gave rise to very few embryos, none of which was in the first cell cycle at the time of dissection. °° Note that we analyzed 46 embryos by time-lapse DIC microscopy in the one-cell stage (A), but only 33 of these were monitored also past the one-cell stage (B).
(PDF)

Table S2 Immunofluorescence analysis of centrosomal and centriolar components in *sas-1* mutant embryos during pronuclear migration or meeting, as well as during mitosis. Embryos were stained for IFA, α-tubulin, as well as the components indicated on the left, counterstained to view DNA and scored after imaging. Embryos were categorized according to how many MTOCs, how many foci of the indicated marker and how many IFA foci they harbored. For example, 2-1-0 indicates an embryo that had two MTOCs, one focus of the indicated marker and no IFA focus. Wild type embryos are almost invariably in the 2-2-2 category, both during pronuclear migration/meeting and mitosis. 1? indicates that there could be a very faint signal but this cannot be fully ascertained. Note that we cannot determine with certainty whether the mitotic embryos are in cycle I or cycle II, since in both cases *sas-1* mutants can assemble a monopolar or a bipolar spindle (see Table S1).
(PDF)

Movie S1 DIC time-lapse movie of a wild type N2 embryo from the time of pronuclear appearance until the 8-cell stage. In all movies, embryos are ∼50 µm-long, the frame rate is 5 seconds, and the movies are played at 10 frames per second. In this and the other movies, time is indicated in min:sec from the beginning of the movie.
(M4V)

Movie S2 DIC time-lapse movie of a *sas-1(t1476)* mutant embryo from pronuclear appearance until cytokinesis after the second cell cycle, undergoing bipolar spindle assembly at the second cycle.
(M4V)

Movie S3 DIC time-lapse movie of a *sas-1(t1476)* mutant embryo from pronuclear appearance until the end of the second cycle, undergoing monopolar spindle assembly at the second cycle.
(M4V)

Movie S4 DIC time-lapse movie of a *sas-1(t1476)* mutant embryo from pronuclear migration until the end of the second cycle, undergoing tripolar spindle assembly at the second cycle.
(M4V)

Movie S5 DIC time-lapse movie of a *sas-1(t1476)*/*eDf2* mutant embryo from pronuclear migration until the end of the first cycle. Note the fast moving yolk granules, indicative of an osmolarity defect.
(M4V)

Movie S6 DIC time-lapse movie of a *sas-1(t1476)* mutant oocyte fertilized by control (*fog-2(q71)*) sperm from pronuclear migration until the 8-cell stage, undergoing monopolar spindle assembly in P2.
(M4V)

Movie S7 DIC time-lapse movie of a *sas-1(RNAi)* mutant embryo with a *sas-1*-like phenotype, from pronuclear migration until the end of the second cycle.
(M4V)

Movie S8 DIC time-lapse movie of a *sas-1(RNAi)* mutant embryo with a stronger phenotype (note the two pronuclei moving slowly towards each other; compare to Movie S2 and Movie S5) from pronuclear appearance until the end of the first cycle.
(M4V)
