# Supplementary figures and images for: SAS-1 Is a C2 Domain Protein Critical for Centriole Integrity in C. elegans
Source: PLoS Genet. 2014 Nov 20;10(11):e1004777. doi: 10.1371/journal.pgen.1004777 (PMC4238951; doi:10.1371/journal.pgen.1004777)

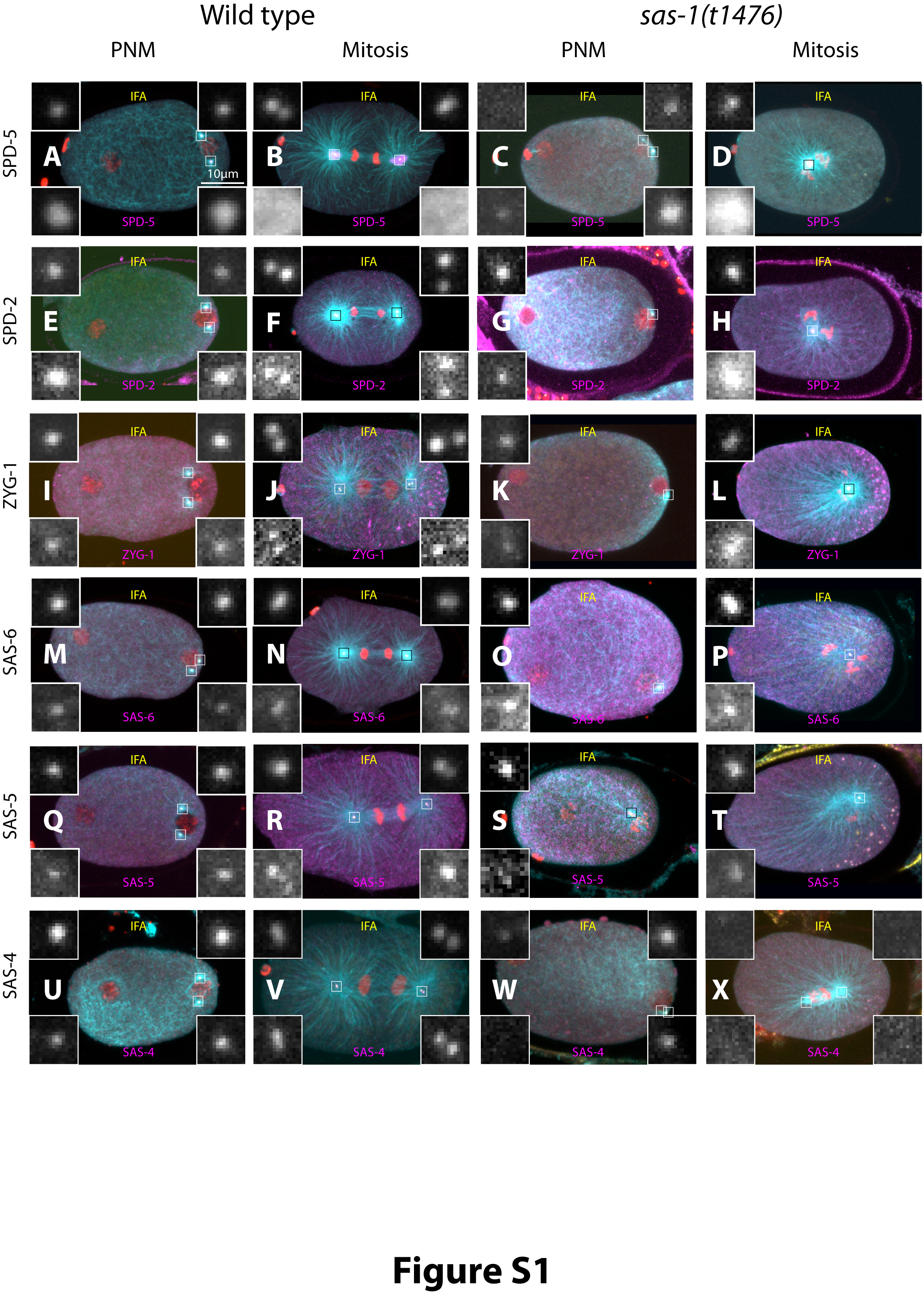

Supplement: Figure S1 — sas-1 mutant embryos harbor only one MTOC at PNM. Wild type (A–B, E–F, I–J, M–N, Q–R, U–V) or sas-1(t1476) (C–D, G–H, K–L, O–P, S–T, W–X) embryos were stained for α-tubulin (cyan), IFA (yellow) and SPD-5 (A–D), SPD-2 (E–H), ZYG-1 (I–L), SAS-6 (M–P), SAS-5 (Q–T) or SAS-4 (U–X) (all magenta). DNA is shown in red. Note that only one MTOC in (C) is IFA positive. Note also that in (W) two IFA foci are visible, but only one harbors SAS-4; the second does not harbor SAS-4 nor is it an MTOC, suggesting that this is a degenerate centriole Note finally that both MTOCs in (X) do not harbor any centriolar marker. See also Table S2. (TIFF) [file pgen.1004777.s001.tiff]

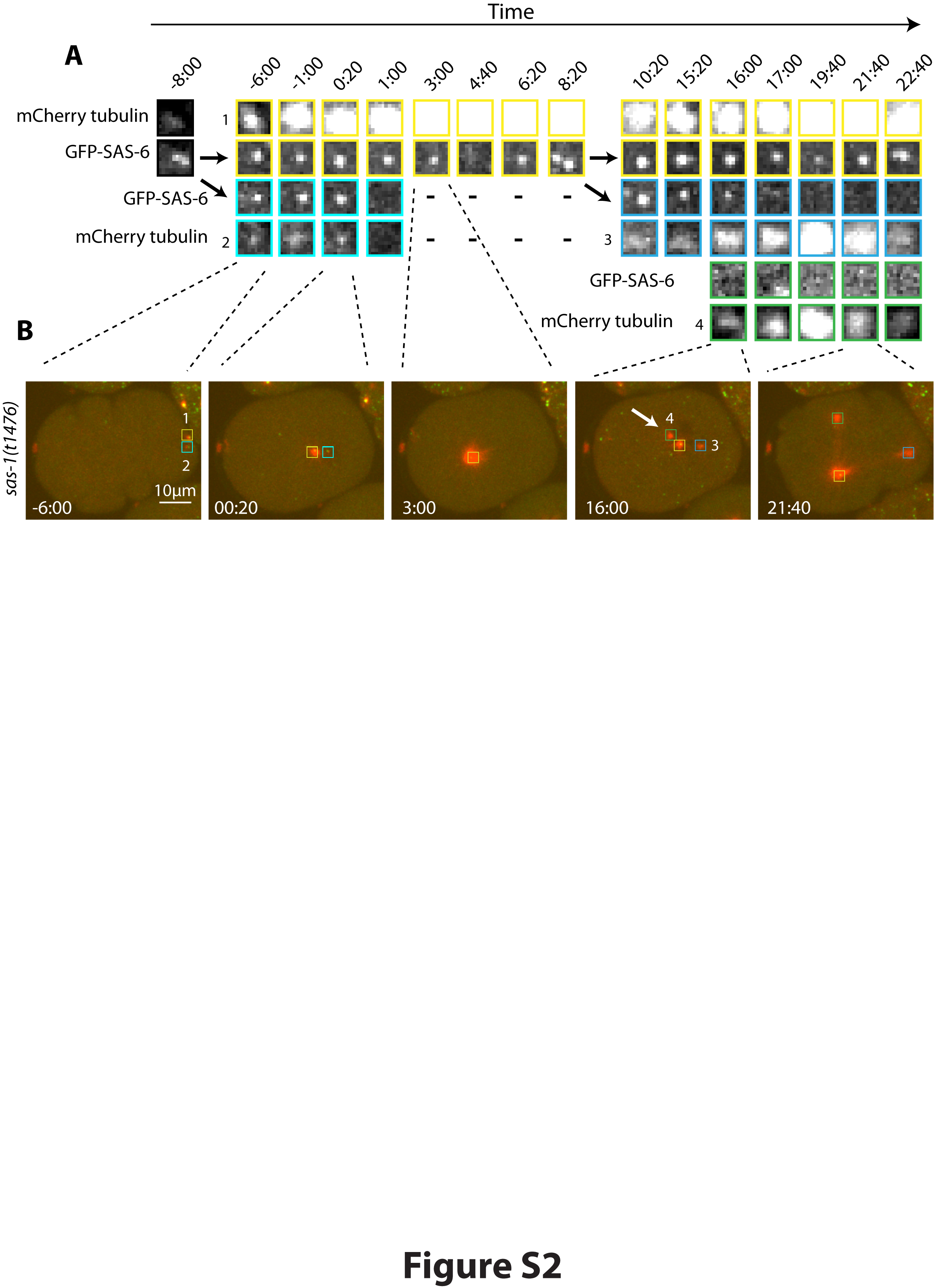

Supplement: Figure S2 — Monitoring the root of tripolar spindle assemblies in sas-1 embryos. sas-1(t1476) homozygous animals expressing GFP-SAS-6 and mCherry-β-tubulin were imaged on a Spinning Disk microscope. (A) Individual MTOCs and corresponding GFP-SAS-6 foci from the embryo in (B) are shown. (B) A representative embryo is shown. After monopolar spindle assembly in the first cycle, a tripolar spindle forms in the second cycle. Note that the embryo harbors two GFP-SAS-6 foci and two MTOCs during pronuclear migration, but looses one before pronuclear meeting. Note also that after the first monopolar mitosis, one MTOC forms without an initial GFP-SAS-6 focus and seems to split off from the already existing MTOC (indicated by white arrow); another MTOC looses GFP-SAS-6. Note that this is in line with the immunofluorescence analysis (Fig. S1C), where some embryos were found to harbor two MTOCs of different sizes. Time is in min and sec, with 00:00 corresponding to pronuclear meeting. (TIFF) [file pgen.1004777.s002.tiff]

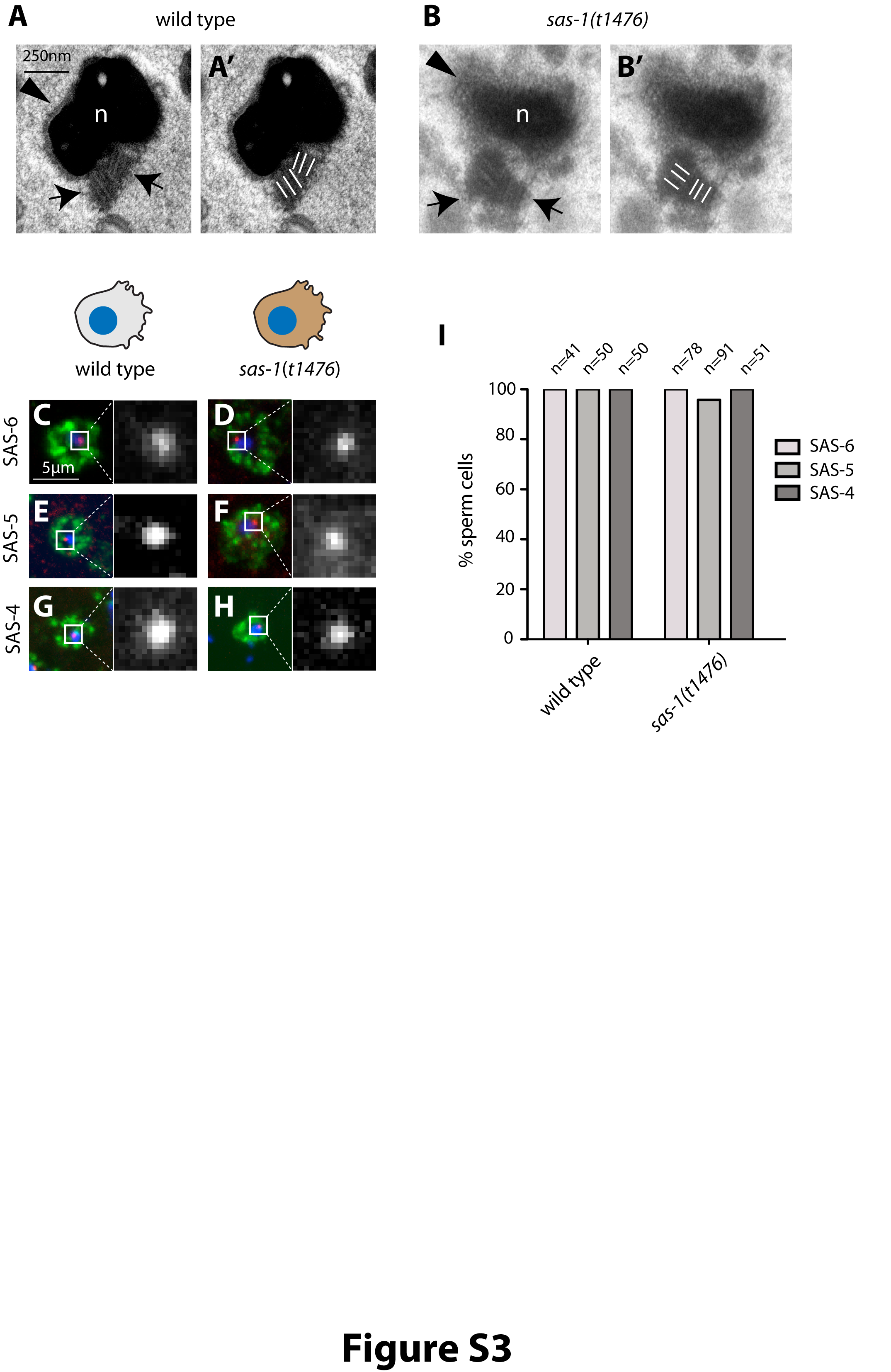

Supplement: Figure S3 — sas-1(t1476) sperm cells have no apparent centriolar defect. (A–B) Electron micrographs of serially sectioned wild type (A) or sas-1(t1476) (B) sperm cells. Centrioles are indicated with arrows. The microtubule blades are schematized in (A′) and (B′). Shown are single 60 nm sections. Due to the difficulties of conducting EM with the minute C. elegans centrioles in sperm cells, one cannot conclude with utmost certainty whether centriole ultrastructure is fully intact in the mutant. Note also that apparent abnormalities were observed occasionally in the perinuclear ring of sas-1(t1476) sperm cells, which was absent or less visible than in the wild type (arrowheads). Furthermore, we often observed extraneous densities elsewhere in the mutant cells (not visible here). 12 wild type and 8 sas-1(t1476) sperm cells coming from one animal each were analyzed by serial sectioning. n = nucleus, arrows indicate centrioles. (C–H) Wild type (C, E, G) or sas-1(t1476) (D, F, H) sperm cells stained for SP-56 (green) to label sperm membranous organelles and with SAS-6 (C–D), SAS-5 (E–F) or SAS-4 (G–H) (red in merge and alone in magnified insets). DNA is shown in blue. (I) Quantification of experiments shown in (C–H). (TIFF) [file pgen.1004777.s003.tiff]

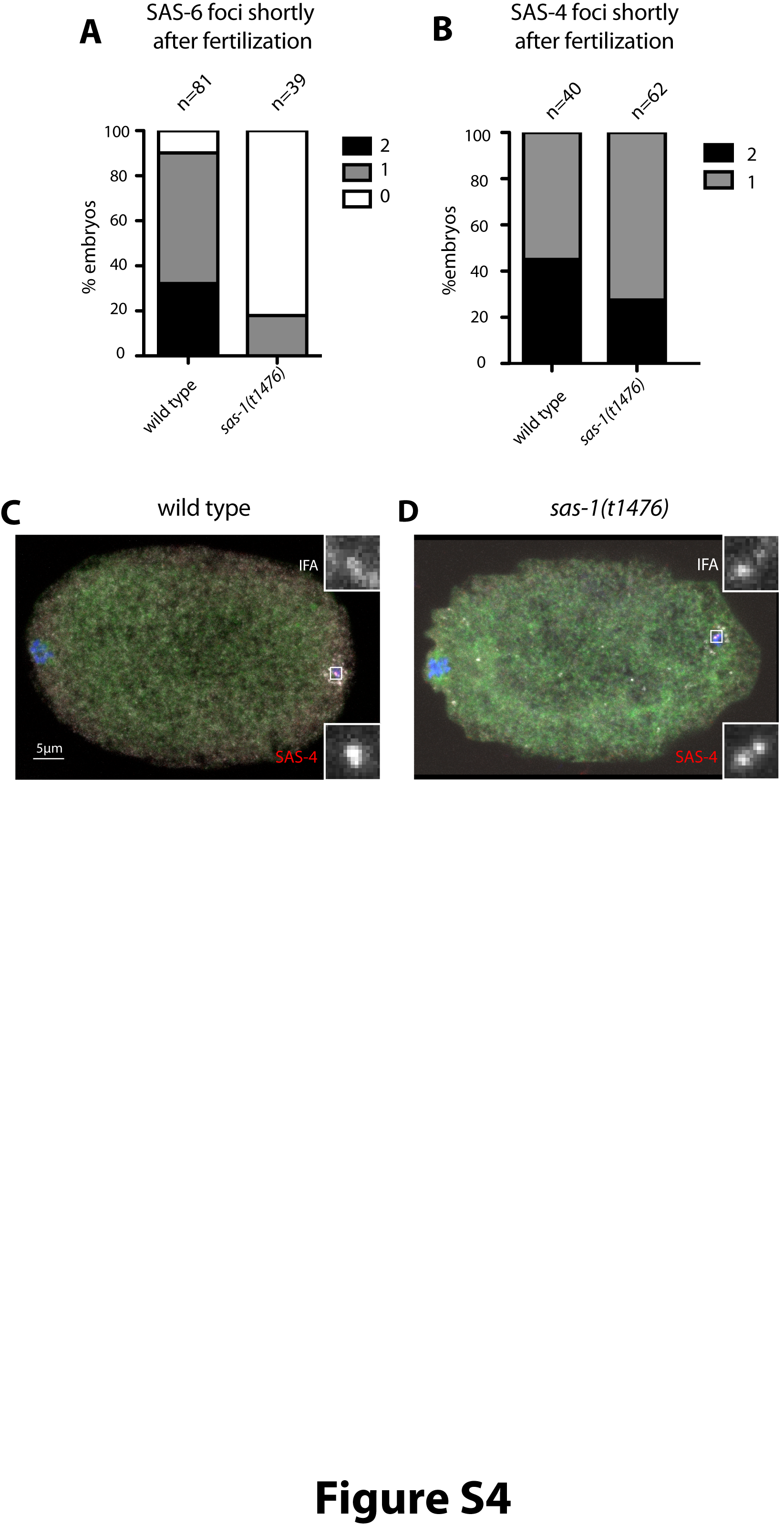

Supplement: Figure S4 — Distribution of paternally contributed SAS-4 and SAS-6 in sas-1 mutant embryos. (A) Quantification of embryos shortly after fertilization stained with SAS-6. (B) Quantification of embryos shortly after fertilization stained with SAS-4 (experiments shown in C–D). Centrioles are often too close to be observed as two separate entities in these early stages. (C–D) Immunostainings of a wild type (C) or sas-1(t1476) (D) embryo for α-tubulin (green), SAS-4 (red), IFA (grey). DNA is shown in blue. Note the two disengaged centrioles in (D). (TIFF) [file pgen.1004777.s004.tiff]

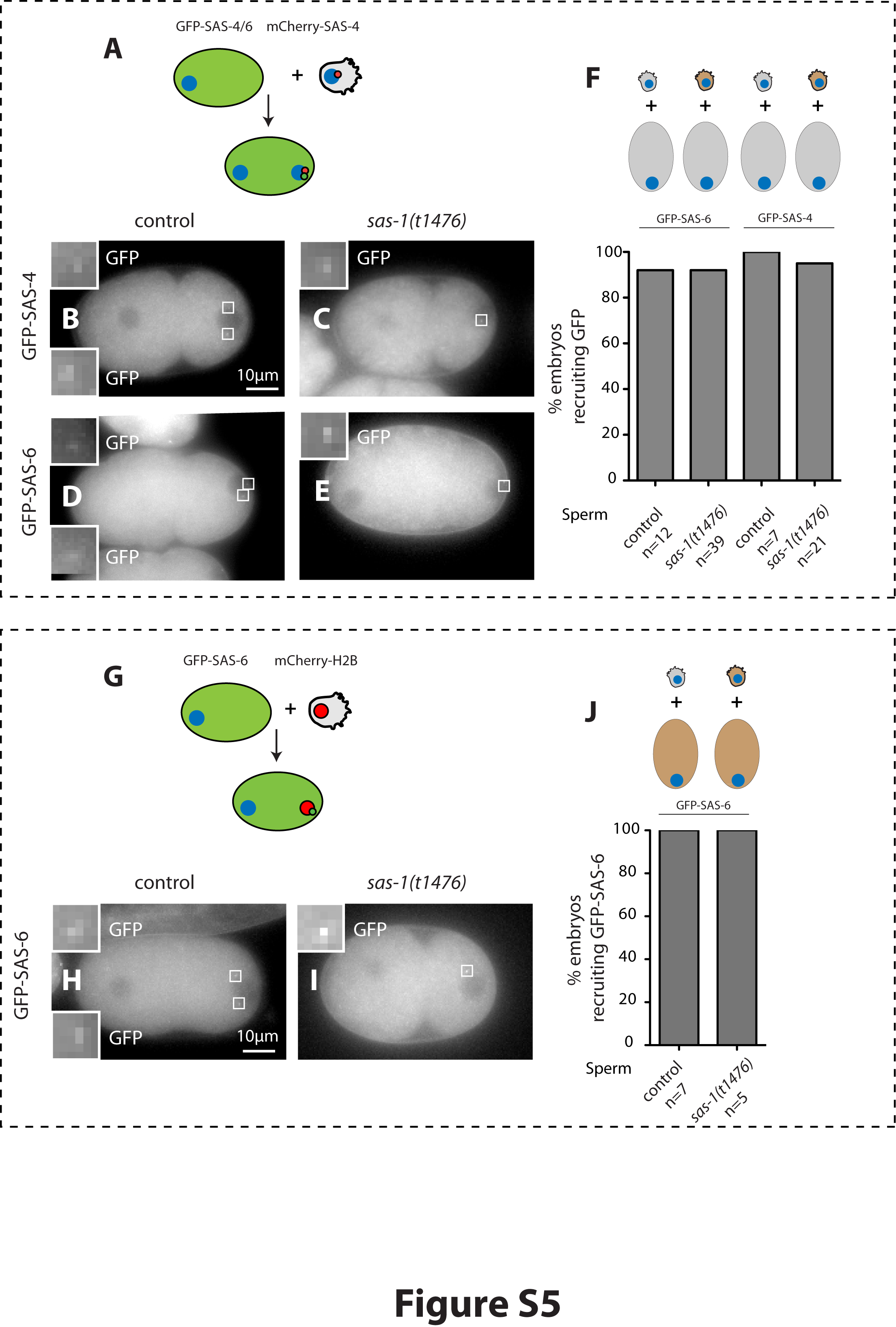

Supplement: Figure S5 — Recruitment of maternal centriolar components is not affected in sas-1 mutant embryos. (A, G) Schematic of experiments performed in (B–E) and (H–I). (B–E) Control (B, D) or sas-1(t1476) (C, E) males expressing mCherry SAS-4 mated to control animals expressing either GFP-SAS-4 (B–C) or GFP-SAS-6 (D–E). Stills of the GFP channel from time-lapse movies are shown; for simplicity, the mCherry signal is not shown. However, we noted that in some sas-1 embryos, the paternal mCherry signal could no longer be detected, in line with the data reported in Fig. 2. (F) Quantification of experiments performed in (B–E). (H–I) Control (H) and sas-1(t1476) (I) males expressing mCherry-H2B were mated to sas-1(t1476) animals expressing GFP-SAS-6. Only animals with mCherry positive paternal DNA were analyzed, since this time we did not mate males to feminized control animals but instead to hermaphrodites. Stills from time-lapse movies are shown. For simplicity, the mCherry signal is not shown. (J) Quantification of experiments performed in (H–I). (TIFF) [file pgen.1004777.s005.tiff]

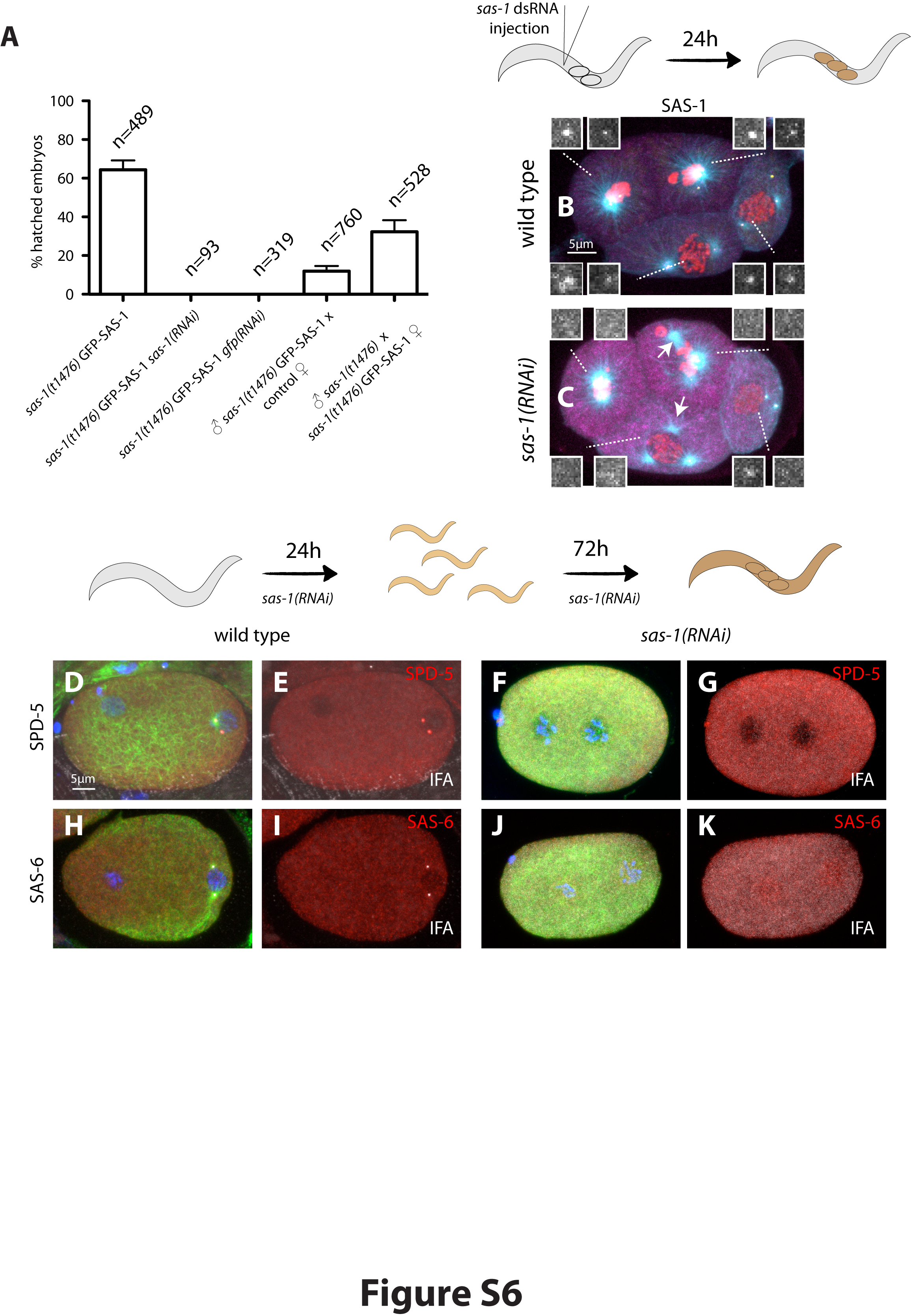

Supplement: Figure S6 — sas-1(RNAi) results in centriole loss and GFP-SAS-1 can rescue the sas-1(t1476) mutant phenotype. (A) Progeny test of indicated conditions, all performed at 24°C. (B–C) Four-cell stage wild type (B) embryos and embryos following injection with sas-1 dsRNA (C) stained for α-tubulin (cyan), SAS-1 (magenta and shown alone in insets) and IFA (yellow). DNA is shown in red. Arrows indicate tripolar spindles; dashed lines indicate the cell from which the insets originate. The vast majority of embryos derived from such injected animals exhibited multipolar spindle assembly in at least one blastomere at the 4-cell stage and thereafter. In the wild type, 81% (n = 43) centrioles were strongly SAS-1 positive and none were SAS-1 negative. In embryos from sas-1 dsRNA injected animals, only 27% (n = 104) were strongly SAS-1 positive (a number that includes the paternally contributed and RNAi resistant sperm centrioles) whereas 58% were SAS-1 negative. Note that the cytoplasmic signal seems to be unspecific, since it is not diminished -but rather increased- in sas-1(RNAi) animals. (D–K) Immunostainings of wild type (D–E, H–I) or sas-1(RNAi) embryos (F–G, J–K) for α-tubulin (green), IFA (grey) and SPD-5 (D–G) or SAS-6 (H–K) (red). DNA is shown in blue. Note the absence of a clear microtubule network in sas-1(RNAi) embryos. N = 11 embryos. (TIFF) [file pgen.1004777.s006.tiff]

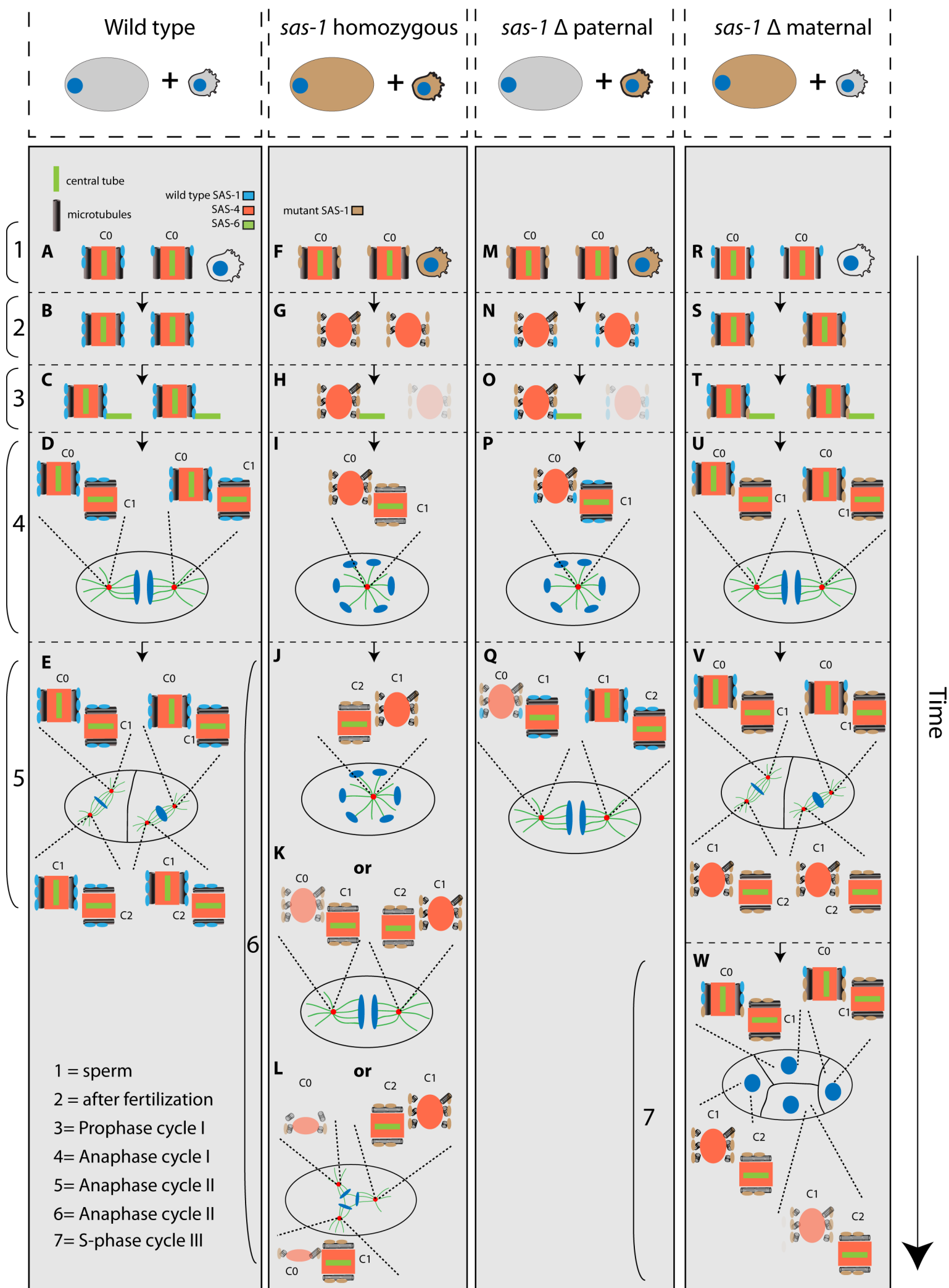

Figure S7

Supplement: Figure S7 — Working model of SAS-1 function and phenotypic consequences of its absence. Simplified centrioles are shown, with SAS-6 (green) building the central tube and SAS-4 (red) the remainder of the structure. Microtubules are shown as grey cylinders or, if abnormal, as wireframe cylinders or cylindrical fragments. C0, centrioles in sperm; C1 centrioles formed next to C0s; C2s centrioles formed next to C1s. Wild type (A–E), sas-1 homozygous animals (F–L), sas-1 mutant sperm fertilizing wild type oocytes (M–Q), wild type centrioles fertilizing sas-1 mutant oocytes (R–W). The different stages are indicated with 1–7. Note that in (G) and (N), two centrioles are still present as judged by SAS-4, but one of them will disappear shortly thereafter. Note that in (I), the C1 centriole has an abnormal microtubule organization that is not recognized as a normal centriole by electron microscopy. After (I), three types of phenotypes can be observed, monopolar (J), bipolar (K) or tripolar (L) spindle assembly, which we explain as follows. For monopolar spindle assembly (J), we surmise that C0 disappeared after fostering formation of C1. Bipolar spindle assembly (K) follows from C0 still being sufficiently present to foster a C1, and the C1 made in the first cycle now fostering a C2; this results in a bipolar spindle, with each pole harboring at least one centriole or centriole-like structure. For tripolar spindle assembly (L), we surmise that C0 breaks apart during mitosis, giving rise to a tripolar configuration. In (N), wild type maternally provided SAS-1 can stabilize centrioles derived from sas-1 mutant sperm to some extent, giving rise to some rescue (see Fig. 1E). In (P), the C1 forms in the presence of wild type maternally provided SAS-1, which presumably leads to formation of a normal procentriole. Accordingly, all divisions thereafter are bipolar (Q). In (V), we hypothesize that the C1s made in (T–U) cannot be stabilized, but can still give rise to C2s (similar to the case in (H [file pgen.1004777.s007.pdf]
