# Supplementary material for: SAS-1 Is a C2 Domain Protein Critical for Centriole Integrity in C. elegans
Source: PLoS Genet. 2014 Nov 20;10(11):e1004777. doi: 10.1371/journal.pgen.1004777 (PMC4238951; doi:10.1371/journal.pgen.1004777)
Supplement: Table S1 — Phenotypes of sas-1 mutant embryos at 24°C. (A–B) Analysis of indicated embryos by time-lapse DIC microscopy in the first cell cycle (A) and in the second cell cycle for the subset of embryos that exhibited monopolar spindle assembly in the first cell cycle (B). (C) Embryonic viability amongst the progeny of animals of the indicated genotypes. We used feminized fog-2(q71) animals that do not produce sperm to rigorously test the paternal contribution of sas-1(t1476). ° Although we dissected 21 sas-1(t1476)/eDf2 animals, we could score the first cell divisions in only six of the resulting embryos, because these animals were either sterile (n = 5) or gave rise to very few embryos, none of which was in the first cell cycle at the time of dissection. °° Note that we analyzed 46 embryos by time-lapse DIC microscopy in the one-cell stage (A), but only 33 of these were monitored also past the one-cell stage (B). (PDF) [file pgen.1004777.s008.pdf]

A

DIC phenotype in first cell cycle

Maternal genotype	Paternal genotype	n	First cycle monopolar
wild type	wild type	10	0
sas-1(t1476)	*sas-1(t1476)*	46	41 (89%)
sas-1(t1521)	*sas-1(t1521)*	16	16 (100%)
sas-1(t1476) / eDf2	*sas-1(t1476) / eDf2*	6	6 (100%)°
wild type	*sas-1(t1476)*	18	14 (78%)
sas-1(t1476)	wild type	6	0
sas-1(t1521)	wild type	14	0

B

DIC phenotype in second cell cycle if first cell cycle monopolar

Maternal genotype	Paternal genotype	n	Second cycle bipolar	Second cycle monopolar	Second cycle tripolar
wild type	wild type	0	0	0	0
sas-1(t1476)	*sas-1(t1476)*	33 °°	24 (73%)	4 (12%)	5 (15%)
sas-1(t1521)	*sas-1(t1521)*	12	5 (42%)	4 (33%)	3 (25%)
sas-1(t1476) / eDf2	*sas-1(t1476) / eDf2*	2	0	2 (100%)	0
wild type	*sas-1(t1476)*	14	14 (100%)	0	0

C

Embryonic viability

Maternal genotype	Paternal genotype	n	Viability (%)
wild type	wild type	257	99
sas-1(t1476)	*sas-1(t1476)*	185	0
sas-1(t1521)	*sas-1(t1521)*	212	0
sas-1(t1476) / eDf2	*sas-1(t1476) / eDf2*	157	0
fog-2(q71)	*sas-1(t1476)*	1435	5

Table S1
