# Supplementary material for: SAS-1 Is a C2 Domain Protein Critical for Centriole Integrity in C. elegans
Source: PLoS Genet. 2014 Nov 20;10(11):e1004777. doi: 10.1371/journal.pgen.1004777 (PMC4238951; doi:10.1371/journal.pgen.1004777)
Supplement: Table S2 — Immunofluorescence analysis of centrosomal and centriolar components in sas-1 mutant embryos during pronuclear migration or meeting, as well as during mitosis. Embryos were stained for IFA, α-tubulin, as well as the components indicated on the left, counterstained to view DNA and scored after imaging. Embryos were categorized according to how many MTOCs, how many foci of the indicated marker and how many IFA foci they harbored. For example, 2-1-0 indicates an embryo that had two MTOCs, one focus of the indicated marker and no IFA focus. Wild type embryos are almost invariably in the 2-2-2 category, both during pronuclear migration/meeting and mitosis. 1? indicates that there could be a very faint signal but this cannot be fully ascertained. Note that we cannot determine with certainty whether the mitotic embryos are in cycle I or cycle II, since in both cases sas-1 mutants can assemble a monopolar or a bipolar spindle (see Table S1). (PDF) [file pgen.1004777.s009.pdf]

Occurrence of centriolar or centrosomal markers during pronuclear migration or meeting (PNM) / Mitosis

IFA<br>indicated marker<br>MTOC	3-2-2	3-2-0	3-1-1	2-3-3	2-2-2	2-2-1	2-1-2	2-1-1	2-1-0	2-0-0	1-2-2	1-2-1	1-1-1	1-1-2	1-0-1	1-1-0	1-1-1?	1-1?-1	0-0-1	1?-1?-1?	1-0-1?	1?-0-0	1-0-0
**SPD-5**						3/0					0/2		3/3										
**SPD-2**					1/0	0/1		0/1	0/1		1/2		4/5	0/1		3/0		1/0				0/1	
**ZYG-1**				0/1			0/1	0/1			0/2		1/2			1/0	1/0				0/1	0/2	
**SAS-6**	0/1		0/1		0/1			0/1			0/1		6/2	0/1	0/2			1/0					
**SAS-5**	0/1										0/2		1/1					1/0	1/0				0/3
**SAS-4**		0/1			0/1			0/1		0/1	1/8	0/1	4/1	2/0						1/0			

- acentriolar MTOC, very faint
- IFA very faint, ZYG-1 faint
- one MTOC very faint, SPD-2 and IFA very faint
- PNM: one MTOC, second centriolar signals of IFA and SPD-2 closeby but very faint
- PNM: both centriolar signals in same MTOC
- focused ZYG-1 signal more widespread than usual
- one MTOC with two centriolar foci
- one MTOC with no centriolar focus
- one IFA focus without ZYG-1 signal, second IFA focus with very faint ZYG-1
- two MTOCs, only one with SPD-2 PCM signal
- two MTOCs with one centriolar focus each, one MTOC with no signal at all
- two weak MTOCs without any centriolar SAS-6

Table S2
